# Phosphoinositide species and filamentous actin formation mediate engulfment by senescent tumor cells

**DOI:** 10.1371/journal.pbio.3001858

**Published:** 2022-10-24

**Authors:** Wesley D. Frey, Ashlyn Y. Anderson, Hyemin Lee, Julie B. Nguyen, Emma L. Cowles, Hua Lu, James G. Jackson

**Affiliations:** Tulane School of Medicine, Department of Biochemistry and Molecular Biology, New Orleans, Louisiana, United States of America; Buck Institute for Research on Aging, UNITED STATES

## Abstract

Cancer cells survive chemotherapy and cause lethal relapse by entering a senescent state that facilitates expression of many phagocytosis/macrophage-related genes that engender a novel cannibalism phenotype. We used biosensors and live-cell imaging to reveal the basic steps and mechanisms of engulfment by senescent human and mouse tumor cells. We show filamentous actin in predator cells was localized to the prey cell throughout the process of engulfment. Biosensors to various phosphoinositide (PI) species revealed increased concentration and distinct localization of predator PI(4) P and PI(4,5)P2 at the prey cell during early stages of engulfment, followed by a transient burst of PI(3) P before and following internalization. PIK3C2B, the kinase responsible for generating PI(3)P, was required for complete engulfment. Inhibition or knockdown of Clathrin, known to associate with PIK3C2B and PI(4,5)P2, severely impaired engulfment. In sum, our data reveal the most fundamental cellular processes of senescent cell engulfment, including the precise localizations and dynamics of actin and PI species throughout the entire process.

## Abstract 2

This study shows that in response to chemotherapy treatment, breast cancer cells that enter a state of cellular senescence employ specific phosphoinositide kinases, actin networks and clathrin to cannibalize and digest neighboring cells, thus accessing nutrients and promoting survival.

## Introduction

Cancer cells can survive chemotherapy and cause lethal relapse if they avoid cell death in conditions of (1) DNA damage and/or mitotic stress caused by therapy; and (2) nutrient deprivation. The breast cancer cells most likely to survive chemotherapy are *TP53* wild type [1,2], and patients with these tumors have very poor survival [3]. We have previously shown that *TP53* wild-type breast cancer cells survive chemotherapy by entering a senescent state that arrests the cell cycle to prevent mitotic catastrophe [4,5] and blocks apoptosis by activity of BCL-XL and/or MCL1 [6,7].

After entering senescence, tumor cells must still support elevated metabolism and survive an environment with unpredictable access to vasculature and nutrient sources. This should be problematic for senescent cells, as they have a high metabolic burden [8–12] that includes the need to produce cytokines and chemokines of the senescence-associated secretory phenotype (SASP) [4,5,13–19]. SASP factors drive protumor phenotypes such as metastasis, stemness, and survival [15,16,20–25]. Senescent cells have increased capacity for autophagy, but this is a limited source for nutrients [26].

We recently identified a novel phenotype of chemotherapy-induced senescent cells: the ability to engulf and break down entire cells in the lysosome [27]. Engulfment occurred after exposure of all 8 cultured cancer cell lines tested to different chemotherapy drugs and in vivo in a syngeneic mouse mammary tumor model. Chemotherapy-treated, senescent cells expressed an entire program of genes related to macrophages and phagocytosis. Engulfment of surrounding cells was unrelated to entosis and occurred at a very high frequency: 15% to 40% of senescent cells in culture had engulfed a neighboring cell. Engulfment conferred a survival advantage [27]. This phenotype had never been previously described and provides an explanation for many characteristics of the senescent cells that contribute to tumor relapse [27].

What is still not known is the mechanism of whole cell engulfment by senescent cells. Various types of engulfment, such as phagocytosis, efferocytosis, or macropinocytosis, are mediated by complex programs that share some features [28,29]. Here we show the program of whole cell engulfment is driven by actin dynamics and generation and localization of phosphoinositide (PI) species that regulate different stages of the engulfment process, and, surprisingly, we found that Clathrin is required for the process.

## Results

### Filamentous actin in predator cells localizes to prey during all stages of engulfment

Actin reorganization is required for fundamental cell processes, including motility and phagocytosis. To monitor dynamics of filamentous (F)-actin formation during senescent cell engulfment, we expressed the biosensor LifeAct-GFP [30], a fusion protein that marks F-actin, in MCF-7 cells that were previously shown to engulf when senescent [6,27]. LifeAct-GFP cells were treated with doxorubicin (DOXO), and interactions with mCherry-expressing, not-treated (NT) cells were cocultured (“DOXO-NT”) and visualized by live-cell imaging and confocal microscopy.

**Fig 1:**
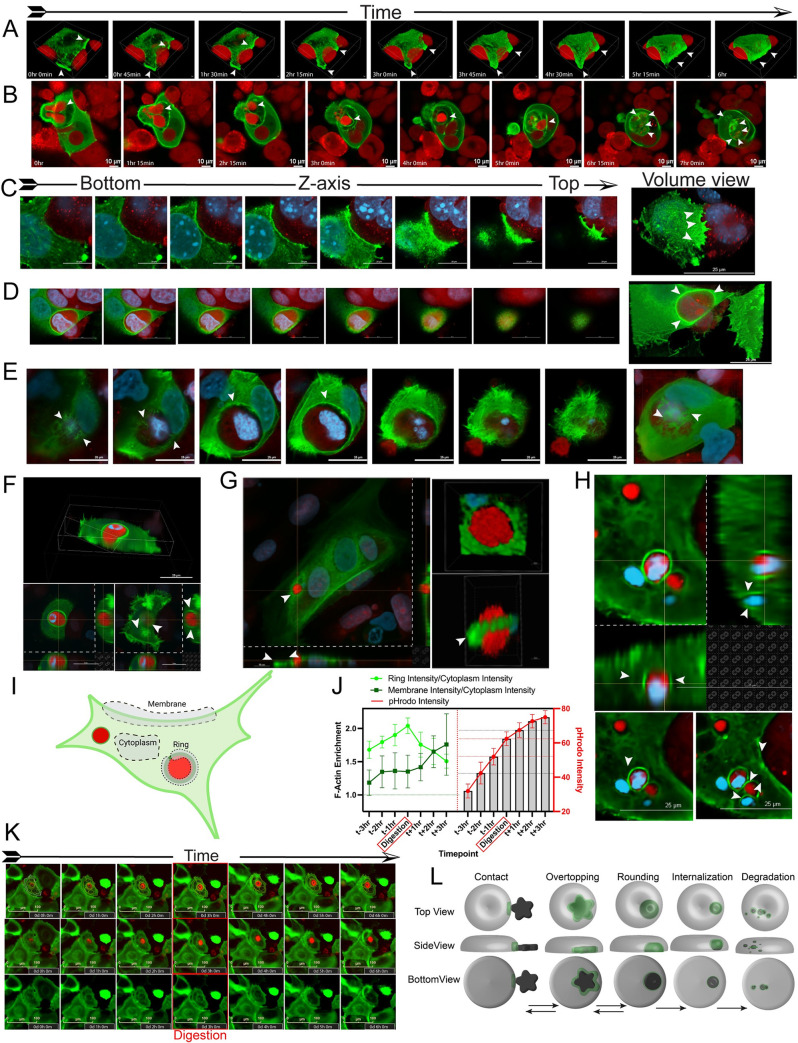
Filamentous actin in predator cells localizes to prey cells throughout the engulfment process. Indicated “predator” MCF-7 cell lines were treated with 250 nM doxorubicin (DOXO) for 24 hours, washed, and plated on indicated not-treated (NT) MCF-7 “prey” cells, creating a “DOXO-NT” culture. Cells were imaged over days 4–8 as noted. (**A**) Volume view reconstruction of time course live-cell confocal imaging of a senescent LifeAct-GFP-MCF-7 cell during contact and partial overtopping of proliferating mCherry-MCF-7 cells on day 7 post-doxorubicin. Concentrated areas of F-actin are indicated by closed arrows, scale bar = 10 μm. (**B**) Volume view reconstruction of time course live-cell confocal imaging of a senescent LifeAct-GFP-MCF-7 cell following engulfment and through partial digestion of proliferating mCherry-MCF-7 cells. The top of the predator cell was removed above the 51.2% axial plane to visualize the internalized mCherry prey cell. Examples of concentrated F-actin are indicated by closed arrows, scale bar = 10 μm. (**C**-**E**) Axial slices along the Z-axis and volume view reconstruction of senescent LifeAct-GFP-MCF-7 cells during engulfment of a proliferating mCherry-MCF-7 cell. (**C**) shows contact and early overtopping, scale bar = 20 μm; (**D**) shows a mostly overtopped prey cell and a volume view with upper part of predator removed as in (**B**) scale bar = 20 μm. (**E**) shows a fully overtopped, engulfed cells with F-actin concentrated in lamellipodia and filipodia above and below the prey, scale bar = 25 μm. 3D volume view is shown from the bottom. (**F**) Volume view reconstruction of a senescent LifeAct-GFP-MCF-7 cell after fully engulfing a proliferating mCherry-MCF-7 cell. The top and side of the predator were removed along axial and sagittal planes to visualize engulfed cell, scale bar = 25 μm. (**G**, **H**) Confocal imaging with z-stack projections shown along right and bottom for senescent LifeAct-GFP-MCF-7 cells after fully engulfing proliferating mCherry-MCF-7 cells. At right of (**G**), volume views to visualize localization of the F-actin ring in the predator with an engulfed mCherry prey cell. Top right is top view, bottom right is side view, scale bar = 10 μm. At bottom of (**H**), axial slices displaying formation of smaller rings around digested portions of prey cell. Scale bar = 25 μm. (**I**) Schematic of a senescent LifeAct-GFP cell with 2 engulfed mCherry prey cells. Shown are example ROIs from the cell membrane, the cytoplasm, and the “ring” around the cell being engulfed. (**J**) Five individual LifeAct-GFP-MCF-7 predator cells were followed during engulfment of pHrodo red stained NT prey cells, and LifeAct-GFP intensity was measured at cytoplasm, nonengulfing membrane, and ring [as depicted in (**I**)] and pHrodo intensity was measured (right). The left graph shows the ratio of LifeAct-GFP intensity in the ring:cytoplasm (light green) and membrane:cytoplasm over 7 hours for 5 cells; error bars represent SEM. The right graph shows pHrodo red intensity over the same time course. Underlying data can be found at S1 Data. (**K**) Time course images of 1 representative cell used to generate data for (**J**). Top: merged green/red channels with ROI used for pixel intensity measurements marked in yellow. Middle: merged green/red channels. Lower: green channel. (**L**) Model of predator cell F-actin localization during the 5 stages of engulfing a prey cell.

Over a time-course of 6 hours covering early stages of engulfment (contact, partial overtopping), confocal microscopy revealed F-actin concentrated in the predator cell at the points of contact with the prey cell, and at the leading edge of lamellar sheets during overtopping of the prey cell (Fig 1A and S1A and S1B Video). Following apparent internalization, F-actin concentrated and dissipated repeatedly around the prey cell(s) during the degradation process of approximately 7 hours (Fig 1B and S2A and S2B Video). F-actin formed structures reminiscent of those present intermittently on lysosomes and endosomes during propulsion through the cell or those associated with phagosomes and required for their mechanical deformation [31,32].

To more precisely localize F-actin in senescent predator cells during various stages of engulfment, we visualized LifeAct-GFP in axial cross sections, 3D volume view reconstructions, and videos. In early stages, we found F-actin concentrated across a broad area of contact with the prey cell, including advancing filapodial structures (Fig 1C and S3 Video). F-actin in the predator cell was tightly associated with the prey cell throughout the overtopping process (Fig 1D and S4 Video). At the time of apparent internalization, F-actin was highly concentrated in filopodial extensions present under and over the top of the prey cell (Fig 1E and S5 Video). Following engulfment and during early digestion as the prey cell repositioned within the predator, F-actin localized to a concentrated “belt-like” structure around the circumference of the prey cell (Fig 1F–1H).

To more quantitatively examine F-actin dynamics during maturation of the phagosome, we stained prey cells with pHrodo, a pH-sensitive dye that fluoresces brightly in the acidic environment of the lysosome. Thus, an increase in pHrodo (red) fluorescence intensity can only occur following maturation and acidification of the phagosome after the prey cell has been internalized by the predator cell. We found that predator F-actin was localized at a ring-like structure associated with the prey cell (as depicted in Fig 1I) before engulfment and increased through engulfment, as pHrodo fluorescence increased (Fig 1J). Following the increase in pHrodo intensity as the prey cell was digested, the F-actin localized at the prey cell diminished sharply but was still maintained at a higher level than in the surrounding cytoplasm (Fig 1J, Ring Intensity/Cytoplasm Intensity). The fluctuating predator F-actin levels localized to the prey cell contrasted with levels observed in nonengulfing areas of the membrane edge, where F-actin was present, but intensity never diminished over the engulfment time-course (Fig 1J, Membrane Intensity/Cytoplasm Intensity). Fig 1K shows a time course of 1 representative LifeAct-GFP-expressing predator cell engulfing a pHrodo (red) stained prey cell. The predator “ring,” “membrane,” and “cytoplasm” areas used to calculate intensity are outlined and diagrammed in Fig 1I.

Similar dynamics were observed for 4226 mammary tumor cell line (S1 Fig). Taken together, the visualization of actin dynamics suggested 5 steps of the engulfment process: predator–prey contact, overtopping of the prey cell (ranging from approximately 10% to 90%), rounding of the prey cell, internalization, and degradation of the prey cell (Fig 1L).

### Phospoinositide species regulate the entire process of whole cell engulfment by senescent cells

PI species interact with and modulate the activity of proteins that reorganize the actin cytoskeleton and are thus critical for the control of actin dynamics in macrophages during phagocytosis of various large targets [33–38].

**Fig 2:**
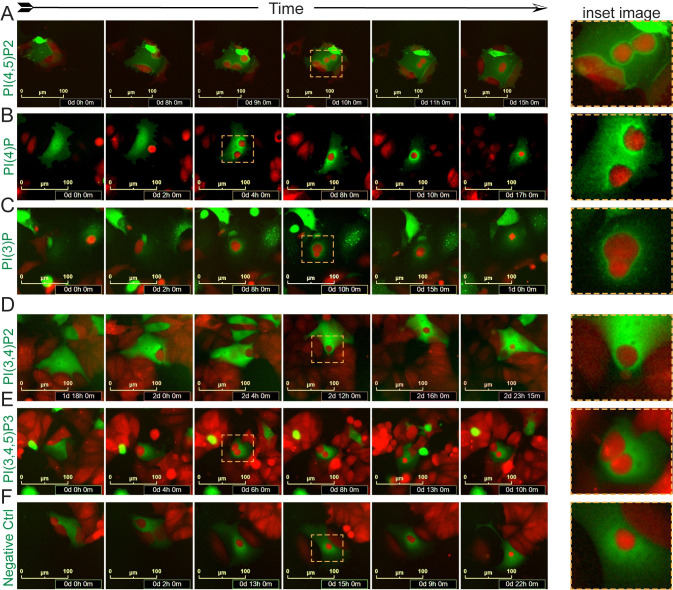
PI biosensors expressed in predator cells reveal PI(4,5)P2, PI(4)P, and PI(3)P, but not PI(3,4,5)P3 or PI(4)P, localize to the prey cells during engulfment. MCF-7 cell lines expressing biosensors that detect indicated PI species were treated with 250 nM doxorubicin for 24 hours, washed, and plated on untreated mCherry-MCF-7 “prey” cells. DOXO-NT cultures were imaged over days 3–8. Time course live-cell imaging of senescent MCF-7 cells that express (**A**) PLCD1-GFP marking PI(4,5)P2; (**B**) P4M-SidMx2-GFP marking PI(4)P; (**C**) 2xFYVE-GFP marking PI(3)P; (**D**) TAPP1-GFP marking PI(3,4)P2; (**E**) BTK-GFP marking PI(3,4,5)P3; (**F**) PLCD1(R40L)-GFP mutant that does not bind PI species (Negative Ctrl), throughout the entire process of engulfing mCherry-MCF-7 cells. Scale bar = 100 μm.

We used specific biosensors to investigate involvement of different PI species in engulfment by senescent cells [39]. Live-cell time course imaging showed PLCD1-GFP [detecting PI(4,5)P2] and SidMx2-GFP [detecting PI(4)P] in the predator cell were closely associated with the prey cell during initial contact, at the overtopping stage, and through the point the predator cell became rounded and was detached from the substrate (Fig 2A and 2B). Both species dissipated in concentration as the digestion process ensued (Fig 2A and 2B). We observed in the predator cell a transient burst of highly concentrated 2xFYVE-GFP [detecting PI(3)P] enveloping the prey after overtopping that remained through rounding and internalization (Fig 2C). Imaging showed biosensors detecting PI(3,4)P2, PI(3,4,5)P3, and a mutant PLCD1-GFP biosensor that does not associate with any PI species (Negative Ctrl) were not concentrated or only diffusely associated with the engulfed cell (Fig 2D–2F), suggesting these species are not involved or are below the sensitivity of detection of their biosensors.

**Fig 3:**
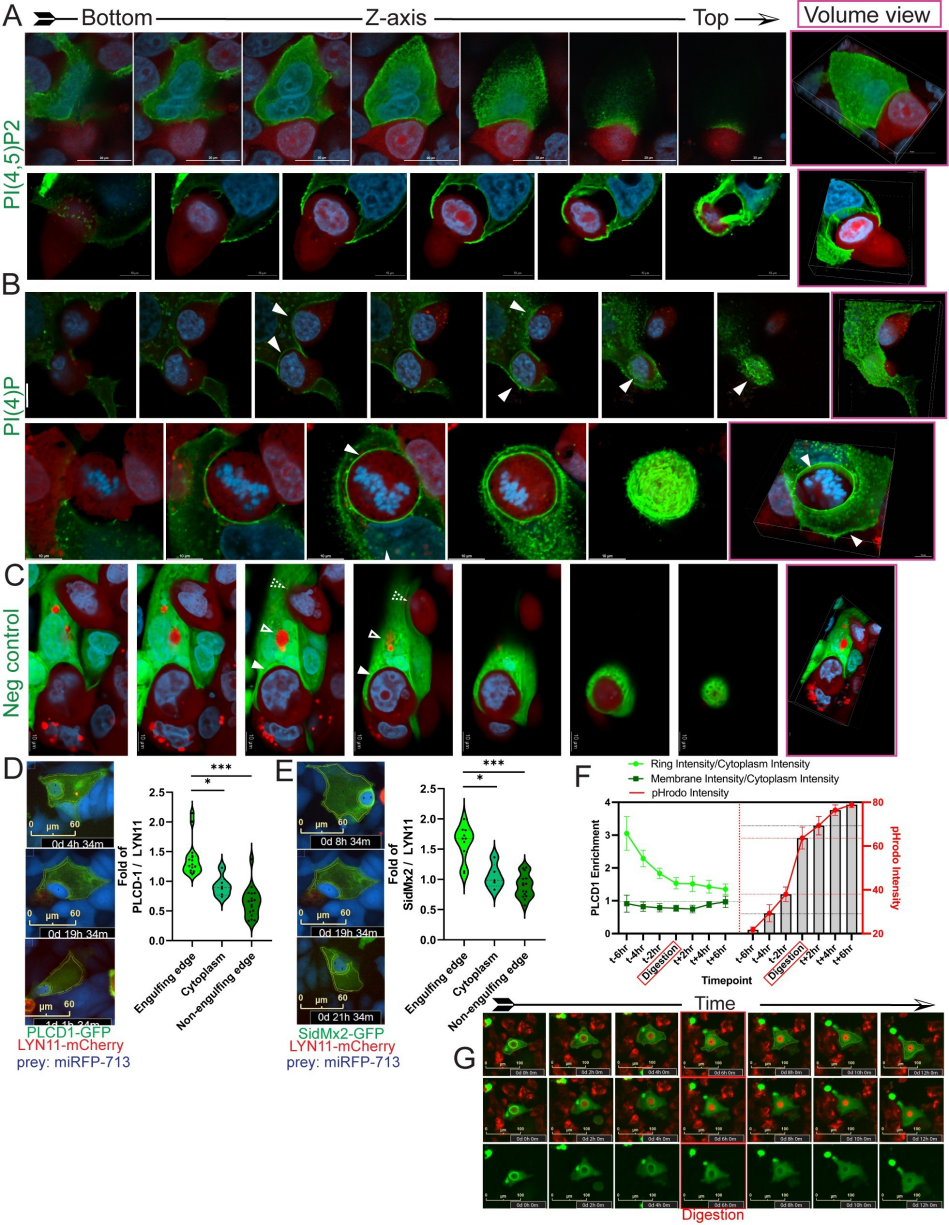
Confocal imaging shows precise localizations of PI(4,5)P2, PI(4)P, and PI(3)P in predator cells during engulfment. Axial slices along the Z-axis and volume view reconstructions of senescent MCF-7 cells, expressing different PI biosensors, during different stages of engulfment in DOXO-NT cultures. (**A**) PLCD1-GFP localization in predator cells during partial (top) and complete (bottom) overtopping of an mCherry-MCF-7 cell (top), scale bar = 20 μm (bottom), scale bar = 10 μm; (**B**) P4M-SidMx2-GFP localization in a predator cell during partial (top) and complete (bottom) overtopping of an mCherry-MCF-7 cell, scale bar = 10 μm. Closed arrows indicate examples of concentrated P4M-SidMx2-GFP localized to lamellipodia advancing over the prey cell. (**C**) Localization of PLCD1(R40L)-GFP mutant that does not bind PI species, during early (closed arrow), mid (dashed arrow), and late stage (open arrow) engulfments of mCherry-MCF-7 cells, scale bar = 10 μm. (**D**) MCF-7 cells coexpressing PLCD1-GFP and LYN11-mCherry (a control that localizes to membranes but is not involved in phagocytic processes) were imaged while engulfing NIR-MCF-7 cells. Left: representative images of 3 cells and the ROI used for quantitation indicated. Right: pixel intensities for the indicated ROI were determined and the ratio of intensity for PLCD1-GFP to LYN11-mCherry was calculated for 2–3 areas of the membrane edge actively contacting the prey cell (engulfing edge), 2–3 areas of the uninvolved membrane (nonengulfing edge), and 1 measurement per cell for the whole cytoplasm. Ratio calculations for 6 individual cells are shown as violin plots with mean and SEM indicated by red dashed line and black dashed line, respectively. Underlying data can be found at S1 Data. (**E**) Ratios as in (**D**) were calculated and shown for MCF-7 cells coexpressing SidMx2-GFP and LYN11-mCherry. Underlying data can be found at S1 Data. (**F**) Five PLCD1-GFP-MCF-7 predator cells were followed during engulfment of pHrodo red stained prey cells, and PLCD1-GFP intensity was measured at cytoplasm, membrane, and ring (as depicted in Fig 1I) and pHrodo intensity was measured (right). The left graph shows the ratio of PLCD1-GFP intensity of the ring:cytoplasm (light green) and membrane:cytoplasm over 12 hours for 5 cells; error bars represent SEM. The right graph shows pHrodo red intensity +/− SEM over the same time course. Underlying data can be found at S1 Data. (**G**) Time course images of 1 representative cell used to generate data for (**F**). Top: merged green/red channels with ROI used for pixel intensity measurements marked in yellow. Middle: merged green/red channels. Lower: green channel. Scale bar = 100 μm. In Fig 4D and 4E, two-way ANOVA was used for analysis, *p*-value < 0.05 = *, *p*-value < 0.01 = **, *p*-value < 0.001 = ***.

To more precisely localize PI species in senescent predator cells during various stages of engulfment, we visualized the biosensors specific for PI(4,5)P2, PI(4)P, and PI(3)P, in axial cross sections, 3D volume view reconstructions, and videos. We found that PI(4,5)P2 was concentrated at the leading edge of the membrane as it contacted prey cells (Fig 3A, upper) and in the cell membrane as it advanced over and around the prey (Fig 3A, lower, and S6 Video). PI(4)P was similarly concentrated at contact early in engulfment (Fig 3B, upper, closed arrow), as the prey was partially overtopped (Fig 3B, upper, and S7 Video), and after complete overtopping and rounding of the prey cell (Fig 3B, lower). Fig 3C shows GFP fused to a mutant PI biosensor varies minimally in concentration within a predator and is only diffusely associated with prey cells during early through late stages of engulfment (Fig 3C). PI(4,5)P2 and PI(4)P both localized to the cell membrane and points of contact at the nonengulfing edges of the cell (Fig 3A and 3B). To determine if these PI species concentrate to a greater extent at sites of engulfment, we quantified the intensity at different regions of the engulfing cell (as depicted in Fig 1I) and compared to another membrane localizing protein that is not involved in engulfment, LYN11 [40,41]. We found the ratio of PLCD1-GFP to LYN11 was greater at the engulfing edge of the phagocytic cup than the nonengulfing edge or in the cytoplasm, suggesting PI(4,5)P2 was preferentially concentrated at sites of engulfment (Fig 3D). Similar results were observed for PI(4)P as detected by SidMx2-GFP (Fig 3E). Quantification over time lapse images showed PI(4,5)P2 dissipated at the prey cell before pHrodo fluorescence, suggesting PI(4,5)P2 was not involved in phagosome maturation post-engulfment (Fig 3F and 3G).

**Fig 4:**
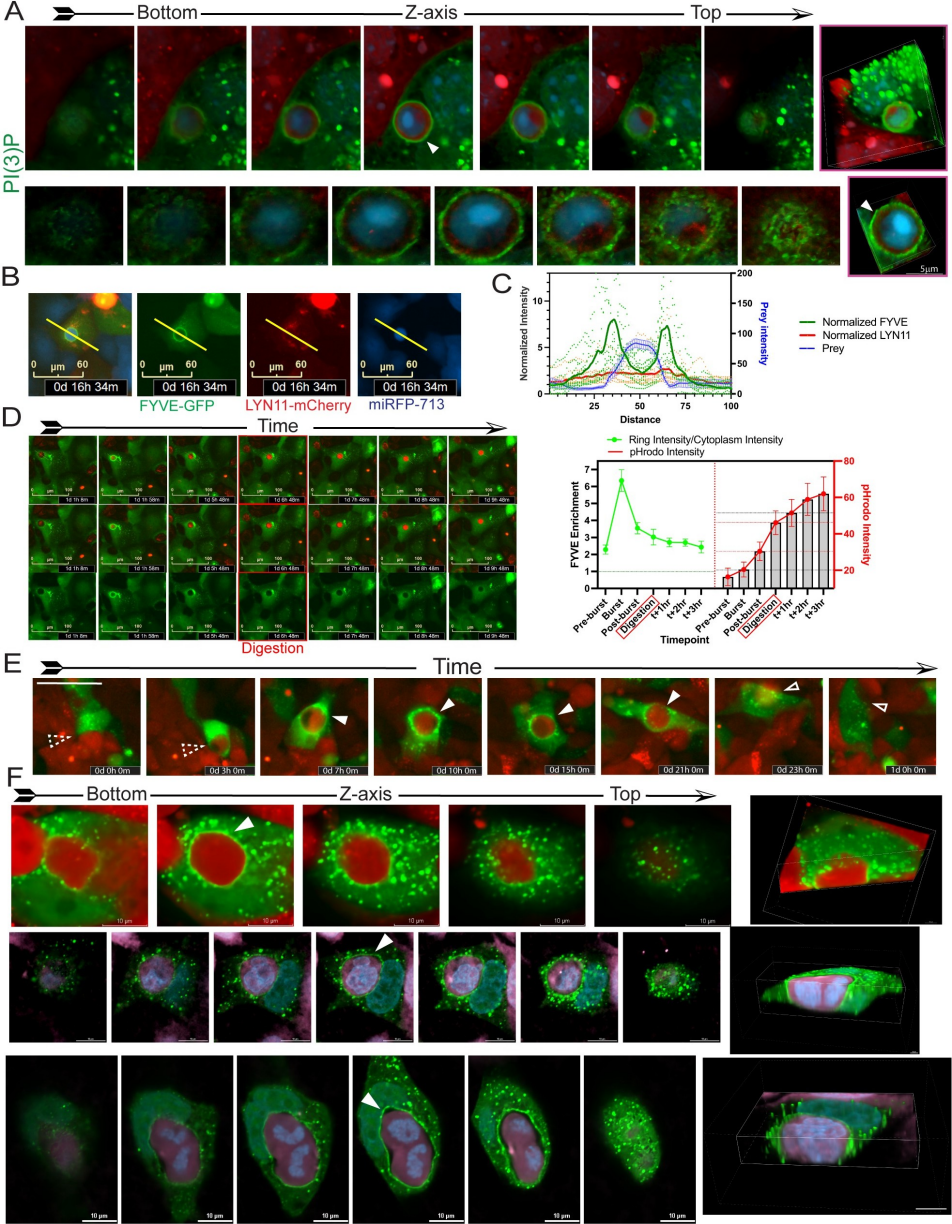
Confocal imaging shows precise localization of PI(3)P in predator cells during engulfment. **(A**) Axial slices along the Z-axis and volume view reconstructions of a senescent MCF-7 cell expressing 2xFYVE-GFP and an mCherry expressing MCF-7 prey cell in a DOXO-NT culture. Top panel scale bar = 10 μm; bottom panel scale bar = 1 μm. (**B**, **C**) Representative image of a 2xFYVE-GFP cell engulfing a NIR-MCF-7 cell. Pixel intensity was determined across the distance of the yellow line and plotted in (**C**) for 5 individual cells. Scale bar = 60 μm. Underlying data can be found at S1 Data. (**D**) Five 2xFYVE-GFP-MCF-7 predator cells were followed during engulfment of pHrodo red stained prey cells, and 2xFYVE-GFP intensity was measured at cytoplasm, membrane, and ring (as depicted in Fig 1I) and pHrodo intensity was measured (right). The right graph shows the ratio of 2xFYVE-GFP intensity of the ring:cytoplasm (light green) over multiple hours surrounding pHrodo intensity increase for 5 cells. Error bars represent SEM. Underlying data can be found at S1 Data. (**D**) Representative images of one 2xFYVE-GFP-MCF-7 predator cell engulfing pHrodo-labeled MCF-7 prey used for Fig 4D (right). ROIs used for quantification are shown in top panel. Scale bar = 100 μm. (**E**) Time course of a 2xFYVE-GFP-MCF-7 cell engulfing an MCF-7-mCherry prey cell. Dashed arrows indicate predator/prey contact; closed arrows indicate the overtopped prey cell with 2xFYVE concentration; open arrows indicate the prey cell no longer overtopped mCherry prey cell. Scale bar = 50 μm. (**F**) Axial slices along the Z-axis and volume view reconstructions of senescent 2xFYVE-GFP-MCF-7 predator cells engulfing mCherry-MCF-7 prey cells. Leftmost image shows the bottom axial slice. Closed arrows indicate 2xFYVE-GFP concentration at the prey cell. Scale bar = 10 μm.

Confocal imaging of 2xFYVE-GFP expressing cells revealed striking images of PI(3)P completely enveloping the prey after rounding (Fig 4A, upper and lower, S8 Video). Quantification of fluorescence intensity in different channels across the predator/prey showed predator cell PI(3)P concentrated at the prey cell far exceeding levels observed for LYN11-mCherry (Fig 4B and 4C). Time lapse imaging with pHrodo-labeled prey cells showed 2xFYVE-GFP was highly concentrated during the transient burst and remained enriched during digestion of the prey cell (Fig 4D and 4E).

In relatively rare instances, predator PI(3)P was observed concentrating at prey cells that were ultimately never engulfed (Fig 4F), suggesting PI(3)P localization was occurring before internalization. Indeed, confocal imaging showed PI(3)P tightly concentrated at prey cells that were clearly still attached to the substrate (Fig 4G). These data contrast to macrophages that localize PI(3)P only *following* phagocytic cup closure and during phagosome maturation [35,42–45], suggesting a novel role for PI(3)P generation in engulfment by senescent cells.

**Fig 5:**
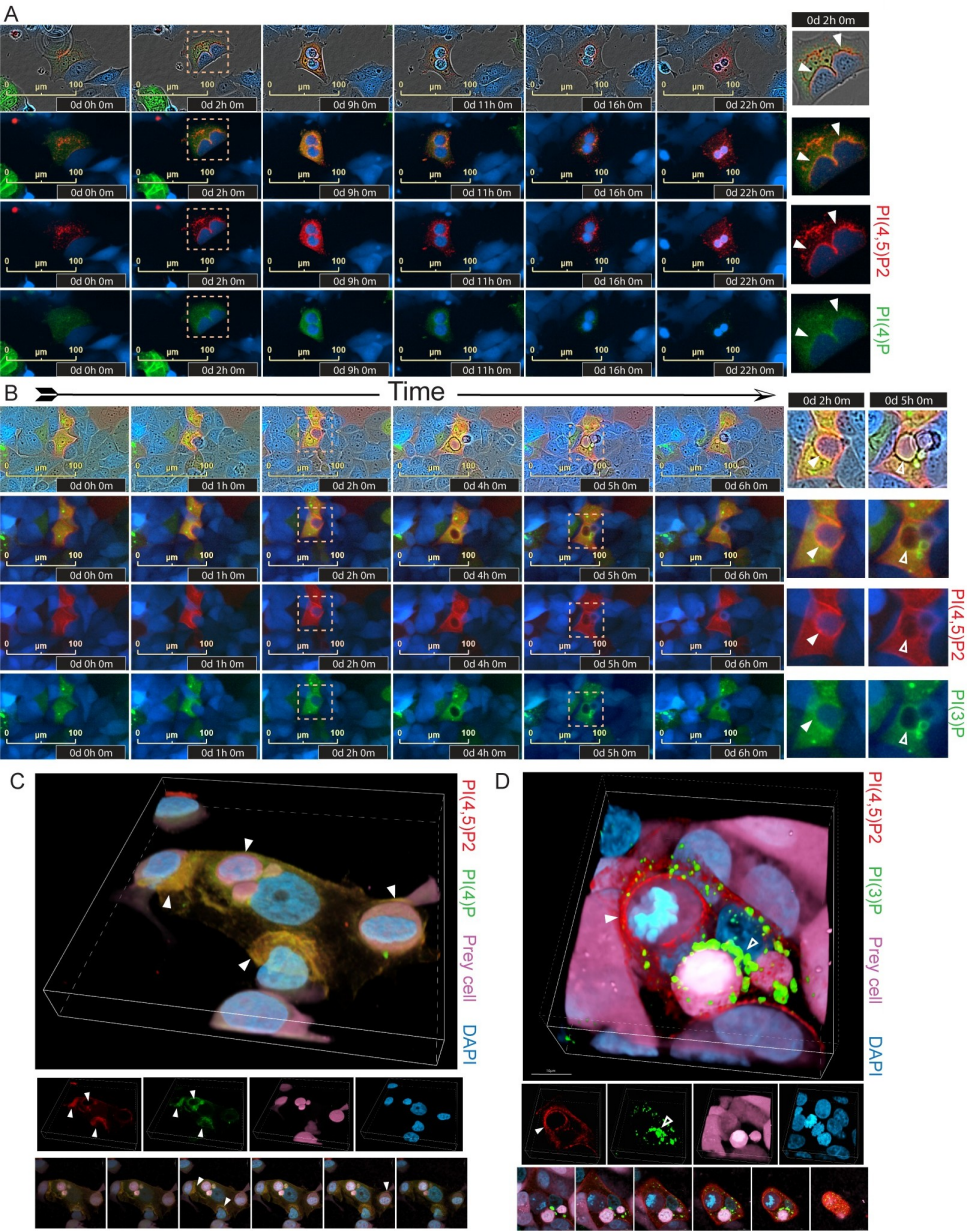
PI(4,5)P2, PI(4)P colocalize at contact through overtopping of prey, then dissipate as PI(3)P localizes tightly around prey during later stages. **(A**) Time course live-cell imaging of a senescent MCF-7 cell that expresses PLCD1-mCherry and P4M-SidMx2-GFP, throughout the entire process of engulfing NIR-MCF-7 prey cells in a DOXO-NT culture. Closed arrows indicate examples PLCD1-mCherry and P4M-SidMx2-GFP colocalization during engulfment. (**B**) Time course live-cell imaging of a senescent MCF-7 cell that expresses PLCD1-mCherry and 2xFYVE-GFP, throughout the entire process of engulfing a NIR-MCF-7 cell. Closed arrows indicate localization of PLCD1-GFP, but not 2xFYVE-mCherry, at mid stage engulfment. Open arrows indicate 2xFYVE-mCherry localization, but not PLCD1-GFP, at late-stage engulfment. Scale bar = 100 μm for A+B. (**C**) Volume view reconstruction of a senescent MCF-7 cell expressing PLCD1-GFP and P4M-SidMx2-mCherry that is engulfing 3 NIR-MCF-7 cells. Middle panels show separate color channels; bottom panel shows axial planes from bottom to top. Closed arrows indicate examples PLCD1-mCherry and P4M-SidMx2-GFP colocalization during engulfment, scale bar = 10 μm. (**D**) Volume view reconstruction of a senescent MCF-7 cell expressing PLCD1-mCherry and 2xFYVE-GFP, which is engulfing 2 NIR-MCF-7 cells. Middle panels show separate color channels; bottom panels show axial planes from bottom to top. Closed arrows indicate localization of PLCD1-mCherry, but not 2xFYVE-GFP, at mid stage engulfment. Open arrows indicate 2xFYVE-mCherry localization, but not PLCD1-GFP, at an internalized cell, scale bar = 10 μm.

We observed accumulation of PI(4,5)P2, PI(4)P, PI(3)P at multiple stages of engulfment (Figs 2–4). To precisely discern differences in their localization and timing during engulfment, we coexpressed biosensors for PI(4,5)P2 and either PI(4)P (Fig 5A) or PI(3)P (Fig 5B) in GFP/mCherry combinations in the same predator cell, and imaged engulfment of near-infrared fluorescent protein (miRFP-713, “NIR”) expressing prey cells. Time-lapse imaging showed near perfect overlap of predator PI(4,5)P2 and PI(4)P during contact and through overtopping of prey cells (Fig 5A, NIR prey cells are blue). Confocal imaging in volume view, of axial slices, and in separated color channels also clearly showed colocalization of PI(4,5)P2 and PI(4)P in a predator cell at different stages of engulfing multiple prey cells (Fig 5C, NIR prey cells are pink). These data suggest similar or identical roles in engulfment and that phosphorylation of the 4 position on the inositol ring is significant.

Further imaging showed PI(4,5)P2 and PI(3)P were localized at entirely different stages of engulfment (Fig 5B and 5D). We found in the predator cell that PI(4,5)P2 was concentrated at the prey cell first, during contact and overtopping, but then dissipated as PI(3)P began to concentrate and localize. We observed very little overlap or colocalization during the transition (Fig 5B). This pattern of localization was evident in confocal imaging in a predator cell engulfing 2 NIR expressing prey cells (pink): one after internalization and one at overtopping. Imaging shows PI(4,5)P2 was concentrated and localized at the overtopped prey cell (Fig 5D) but not the internalized prey cell, while PI(3)P was concentrated and localized to the internalized, rounded prey cell (Fig 5D, green localization, open arrow). Similar PI species localizations were observed in 4226 cell line (S2 Fig). Dynamic localization of different PI species throughout the process of engulfment is depicted in S3 Fig.

### PIK3C2B facilitates internalization

**Fig 6:**
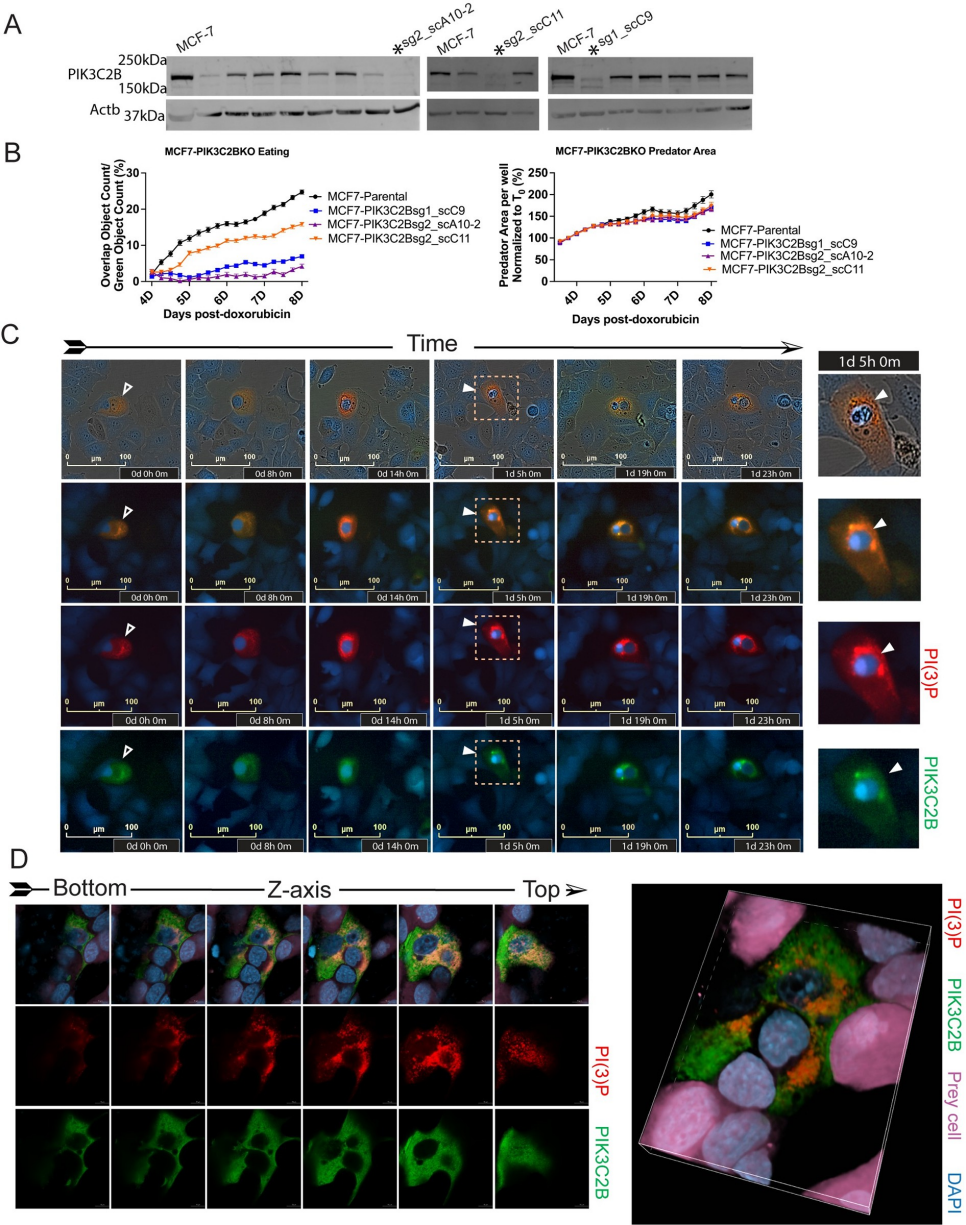
Predator cell PIK3C2B colocalizes with PI(3)P during engulfment and is required for complete internalization. (**A**) CRISPR-Cas9 single-cell clone PIK3C2B knockouts of MCF-7 were screened by immunoblot. Clones indicated by asterisk were chosen for further testing. (**B**) Predator cell engulfment rates (left) and confluency as a measure of viability (right) for senescent MCF-7 parental cells and 3 PIK3C2B knockout clones were determined by time course imaging in a DOXO-NT culture. Underlying data can be found at S1 Data. (**C**) Time course live-cell imaging of a senescent MCF-7 cell expressing 2xFYVE-mCherry and PIK3C2B-GFP throughout the entire process of engulfing an NIR-MCF-7 cell, scale bar = 100 μm. (**D**) Left: Merged (top) and separate (middle, lower) color channels of axial planes from bottom to top of a senescent MCF-7 cell expressing 2xFYVE-mCherry and PIK3C2B-GFP, engulfing NIR-MCF-7 cells. Right, volume view reconstruction of the same image, scale bar = 10 μm.

Based on imaging data (Figs 2–5), the PI species most likely to play a nonredundant, critical role for the final, irreversible stages of engulfment is PI(3)P. This species is generated by 4 different kinases [46], and we used CRISPR-Cas9 to derive single-cell knockout clones of each (Figs 6A and S4A). Engulfment rates determined on IncuCyte (Materials and methods, S6 Fig), and viability of predator cells with knockout of *PIK3C2G*, *PIK3C3*, *PIK3C2A* were not reduced (S4B Fig). PIK3C2B has a well-described role in cancer biology, therapy resistance, cell motility, and cytoskeletal dynamics [47–54]. We found that cells deficient in *PIK3C2B* were relatively normal and were not more sensitive to the chemotherapy (Fig 6B, right, and S4D Fig, right). We found multiple PIK3C2B knockout clones had reduced rates of whole cell engulfment (Fig 6B, left, and S4D Fig, left). In PIK3C2B knockout cell lines, we observed that predators formed lamellar projections, made contact with and successfully overtopped prey (S4E and S4F Fig), but were less likely to complete engulfment of adherent prey (Fig 6B).

Time course live cell imaging showed PIK3C2B-GFP fusion protein began to accumulate near prey cells during overtopping and increased in concentration through rounding and internalization of the prey and was partially colocalized with PI(3)P during much of this process (Fig 6C). Confocal microscopy showed both PIK3C2B-GFP and PI(3)P were present independently in the predator and did not completely overlap in localization, but significant areas of overlap were observed at the site of an overtopped prey cell (Fig 6D). In both time-lapse and confocal imaging, we noted the cytoplasmic PIK3C2B protein was diffusely present and did not associate as tightly to prey cells as the biosensors that detected membrane bound PI species.

PI3 kinase activity and generation of PI(3,4,5)P3 plays a critical role in phagocytosis of large substrates [55, 56], and thus it was surprising to observe no localization during senescent cell engulfment (Fig 2). To determine if PI(3,4,5)P3 contributes to engulfment in our senescent cells without actually localizing to sites of engulfment, we treated cells with 2 selective PI3 kinase inhibitors, BKM-120 (targeting p110α/β/δ/γ) [57] and GDC-0941 (targeting p110α/δ) [58]. Because the entire culture of predator/prey is exposed to drug, any observed change in engulfment could be due to drug effects on the proliferating prey cell, and not the senescent predator cell. To mitigate this, we assayed engulfment of senescent predator/senescent prey cells (S5 Fig, DOXO-DOXO) in cultures as we have previously [27].

Using concentrations that were mostly nontoxic to the cells (S5A and S5B Fig), we observed no decrease in predator engulfment rates for MCF-7 or 4226 mammary tumor cells (S5C and S5D Fig). MCF-7 cells were more sensitive to the drugs (S5C Fig), but at concentrations that were beginning to cause cell death (S5C Fig, lower), more engulfment was actually observed (S5C Fig, upper). It is possible this could be the engulfment of dead/dying cells through efferocytosis by the senescent predator cells, as has been previously observed [27]. Nevertheless, we did not observe reduced engulfment rates in MCF-7 or 4226 cell lines treated with potent inhibitors of PI3 kinase. Taken together with localization data (Fig 2), these data suggest generation of PI(3,4,5)P3 is not critical for senescent cells to engulf other whole cells.

### Clathrin is involved in senescent cell engulfment

**Fig 7:**
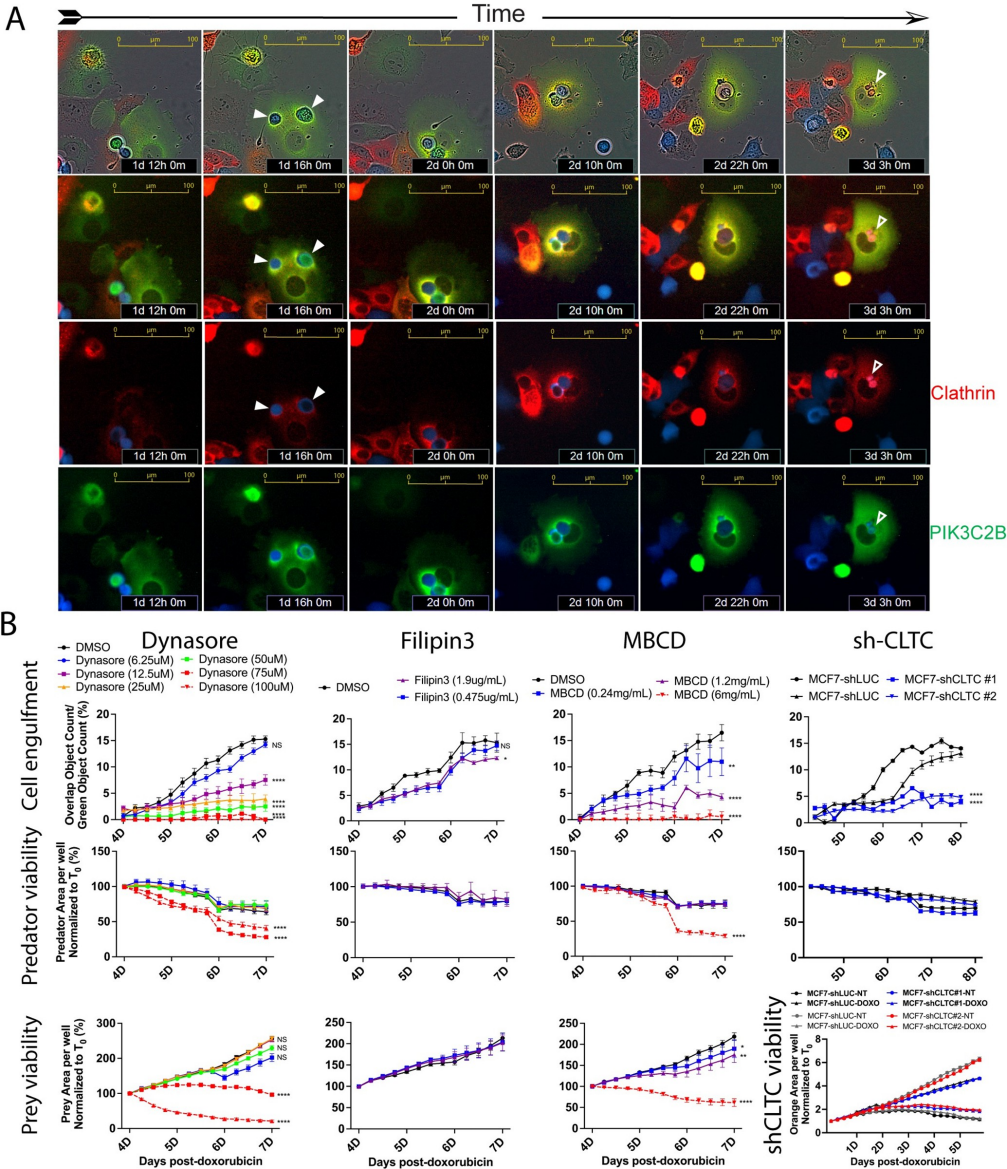
Clathrin relocalizes to sites of engulfment and is required for efficient completion of internalization. **(A**) Time course live-cell imaging of a senescent MCF-7 cell expressing CLTC-mCherry and PIK3C2B-GFP, throughout the entire process of engulfing an NIR-MCF-7 prey cell in a DOXO-NT culture. Scale bar = 100 μm. (**B**) Predator cell engulfment rates (upper) and confluency (lower) in DOXO-NT cultures were determined for senescent GFP-MCF-7 cells that were treated with indicated drugs or made to express an shRNA construct targeting *CLTC*. Concentrations of Dynasore, Filipin3, and MBCD producing toxicity are displayed as red lines (Dynasore 75 uM, 100 uM and MBCD 6 mg/mL). Underlying data can be found at S1 Data.

After finding evident involvement of PIK3C2B in senescent cell engulfment, we investigated potential downstream mechanisms. Interestingly, PIK3C2B directly associates and colocalizes with Clathrin [59], a protein known to create cages for endocytosis and facilitate nucleation of F-actin during engulfment of substrates ranging from small to large [60,61]. Components of the Clathrin complex also associate with PI(4,5)P2, which we found highly enriched at sites of engulfment (Figs 2–4) [62,63]. In senescent cells not actively engulfing or contacting other cells, Clathrin was localized to its typical, perinuclear space (Fig 7A). When a prey cell was overtopped by a predator cell, Clathrin concentrated and localized around and over the prey, colocalized with PIK3C2B (Fig 7A, closed arrows). Following internalization, as the prey cells were digested, Clathrin was redistributed back to the perinuclear space (Fig 7A, open arrows). Next, we treated cells with 3 drugs that all have in common inhibition of Clathrin-mediated endocytosis (CME) [64] (Dynasore [65], Filipin3 [66], MBCD [67]). While these drugs are not highly specific to Clathrin, we did observe reduced engulfment rates at the day 8 posttreatment time point (Figs 7B and S7A). Filipin3 treatment decreased engulfment by 23.7% and 61.8% in MCF-7 and 4226 cells, respectively. Inhibition of dynamin using Dynasore decreased engulfment by 52% in MCF-7 cells and by 58.1% in 4226 cells. MBCD reduced engulfment by 74% in MCF-7 and 55.9% in 4226 (Figs 7 and S7A). Though we identified doses of drugs that were toxic to predator and prey cells (Fig 7B, red lines), engulfment was still inhibited at a wide range of well-tolerated concentrations (Figs 7B and S7A). To rule out drug effects on prey cells (rather than on predator cell engulfment mechanisms), we treated cultures of senescent predator/senescent prey (DOXO-DOXO) cells, and, in these conditions, similar reductions in engulfment rate were observed (S7B Fig). Because, in addition to Clathrin, these drugs can inhibit other proteins, we used shRNA to knock down *CLTC* (S7C Fig). *CLTC* knockdown dramatically reduced rates of engulfment (71.5% in MCF-7 and C60.6% in MPE600), supporting the results with the CME inhibitors (Figs 7B and S7D, shCLTC).

## Discussion

Our group previously showed breast tumor cells that enter senescence to avoid apoptosis and mitotic catastrophe after chemotherapy express many genes related to different mechanisms of engulfment [27]. These include genes related to phagocytosis, efferocytosis, macropinocytosis, and endocytosis, thus resulting in a novel phenotype that includes whole cell cannibalism. Here, we determined the fundamental mechanisms mediating the process by which a senescent tumor cells cannibalizes a healthy neighboring cell. We defined 5 basic steps in the process (**Fig 1L**), and we show F-actin is involved throughout the process, from contact to relatively late in digestion. Recent studies have described 2 distinct modes of actin-mediated macrophage engulfment, lamellipodial and filopodial, depending on the spatial constriction or crowding of the cells [68]. As senescent predator cells contacted and overtopped prey cells, we observed formation of both lamellar sheets and elongated filopodial extensions that were rich in F-actin (Fig 1). However, the predominant type of engulfment in senescent breast cancer cells appears to be the sheet-like overtopping shown in Fig 1A. As fully engulfed cells were processed, F-actin was highly concentrated around the circumference of the engulfed cell. As the prey cell became smaller and broken down from one large vesicle to multiple smaller ones, concentrated F-actin structures formed, disappeared, and reformed continuously (Fig 1B and S2 Video). These observations are highly reminiscent of transient actin structures formed in macrophages that mediate mechanical breakdown (described as “chewing”) of internalized bacteria or red blood cells [32,69].

PI species mediate numerous cell processes, including engulfments such as phagocytosis. During engulfment, PI(4)P and PI(4,5)P2 become concentrated and precisely localized at the prey cell. The fact these 2 species appear to have overlapping localization suggests possible redundancy that could, perhaps, extend to the kinases responsible for generating them. PI(3)P was the only species detected at the critical stages of rounding and internalization, and at least 1 kinase responsible for generating PI(3)P was required for efficient engulfment. Distinct from other forms of phagocytosis [35], we observed enriched localization of predator PI(3)P at prey cells before internalization (Fig 4D–4G). Further, while PI3 kinase activity and generation of PI(3,4,5)P3 is critical for phagocytosis of large substrates by macrophages [55,56], in senescent cells, PI(3,4,5)P3 did not localize to sites of engulfment, and phagocytosis occurred efficiently in the presence of PI3 kinase inhibitors (S5 Fig).

While our cells with knockout of PIK3C2B had reduced rates of engulfment, we cannot rule out involvement or compensation by the other kinases responsible for generating PI(3)P: PIK3C3, PIK3C2A, and PIK3C2G [46]. Knockout of these kinases, however, had no effect on engulfment, suggesting a primary role for PIK3C2B. It is also possible the kinase(s) necessary and/or sufficient for PI(3)P generation are tissue or cell type specific.

Identifying Clathrin involvement was surprising but also made sense in light of evident roles for PI(4,5)P2 and PIK3C2B, both known to interact with Clathrin [59]. Roles for Clathrin in mediating engulfment of larger targets are emerging [61], and Clathrin involvement in engulfment by senescent cells may be related to its role as an actin network organizer rather than classic, coated pit endocytosis [61]. Recently, Clathrin was also shown to be involved in the final stages of phagocytosis: resolution of phagosomes [70].

In sum, we present the first data demonstrating the mechanistic underpinnings of the remarkable cell cannibalism phenotype of breast tumor cells that enter senescence to survive chemotherapy. Understanding how senescent cells engulf could inform strategies to eliminate them, thus improving treatments in the cancer most difficult to eradicate.

## Materials and methods

### Cell culture

MCF-7 cells were from ATCC and cultured in complete EMEM media (ATCC). MPE600 cells were a gift of Joe Gray (Oregon Health Sciences University) and were cultured in complete DMEM media. 4226 cells, created from a spontaneous MMTV-*Wnt1* tumor, were cultured in complete DMEM and have been previously described [6,27].

### Vector construction

Plasmids for tracking PI species were from Addgene. Specifically, mCherry-Clathrin LC-15 (Addgene #55019) [71]; PLCD1(PH)-mCherry (Addgene #36075) [72]; PH-PLCD1-GFP (Addgene #51407), PH-PLCD1(R40L)-GFP (Addgene #51408) [73]; pLentiLifeACT-EGFP BlastR (Addgene #84383) [74]; PH-Btk-GFP (Addgene #51463), GFP-PH-TAPP1 (Addgene #161985); and pLenti-EGFP-P4M-SidMx2 (Addgene #136997), pLenti-EGFP-2xFYVE (Addgene #136996) were gifts from Ken-Ichi Takemaru. Plasmids were also constructed by Vectorbuilder: VB200917 is a lenti-mCherry-shCLTC containing U6-driven shRNA sequence 5′-CGTGTTCTTGTAACCTTTATT-3′. VB200917 is a lenti-mCherry-shLuciferase containing U6-driven shRNA sequence 5′- ATGTTTACTACACTCGGATAT-3′ and was used to create lentivirus to infect MCF7, 4226, and MPE600 cell lines. Infected cells were sorted for mCherry using FACS as above and tested for engulfment. Human PIK3C2B-GFP fusion overexpression vector is a lentiviral CMV-driven plasmid from VectorBuilder. miRFP-713 (near-infrared fluorescent protein) overexpression vector is a lentiviral EFS-driven plasmid from VectorBuilder. 2xFYVE-domain-mCherry fusion protein is lentiviral plasmid from VectorBuilder. Lentiviral EFS-driven LYN11 mCherry fusion protein construct was also produced by VectorBuilder.

### Lentiviral infections

Lentivirus was produced using 293T cells and a standard Lipofectamine 2000 protocol according to the manufacturer’s instructions (Thermo Fisher Scientific). Viral supernatants were collected 48 to 72 hours after transfection and filtered (0.45 μm). MCF-7 cells were plated at 166,000 cells in a 6-well plate, then spinfected with the filtered viral supernatant at 1,100 x *g* for 20 minutes the following day. Positive cells were FACS sorted using a BD FACSAriaII as previously published [5].

### CRISPR-mediated gene deletion

Guides for CRISPR-mediated gene editing (human target sequences: PIK3C2Bsg1 ATGGCGTATGTCATCAACG and PIK3C2Bsg2 CATGGCGTATGTCATCAAC) (Mouse targeting sequences: Pik3c2bsg2 GACCTGTGGTCGAAAGCTCC, Pik3c2bsg3 ATCAAGTTCACCAGCCCCCC) were designed to target regions of the PI3Ka domain of *PIK3C2B*. These guides were hybridized to their antisense oligos and ligated into BsmBI-digested pLentiCRISPRV2-mCherry [Addgene #75161] backbone. Resultant vectors were sequence verified by sanger sequencing via Genewiz. Lentivirus was created as described above and used for infection of MCF7. Infected cells were sorted for mCherry using a BD FACSAriaII cell sorter and serially diluted giving rise to single-cell clones. Individual clones were grown out and tested for PIK3C2B expression by western blot. Clones confirmed to have homozygous deletions of PIK3C2B were then used for engulfment assays. Single-cell clones with knockouts of *PIk3C2A*, *PIK3C3*, and *PIK3C2G* in MCF7 were created using the same backbone and targeting strategy. The target sequences used for *PIK3C2A* were PIK3C2Asg1 GGATCCTCTAAGTAAGCCTA, PIK3C2Asg2 CAATATAATGCTTCGAAGCA. The target sequence used for *PIK3C3* was PIK3C3sg3 GGCTGAAGTTCTTGATACAG. The target sequences used for *PIK3C2G* were PIK3C2Gsg2 AATGGCGATGAATCTTTGCT, PIK3C2Gsg3 TGTGACGGTCACATACTCCC.

### Drug treatments

Doxorubicin was from Santa Cruz Biotech (SC-200923A), resuspended in water at a concentration of 10 mg/mL, and then diluted in PBS to 50 μg/mL and used at concentrations indicated. Filipin III (Filipin3) was from Sigma (Cat# F4767). Dynasore was from Abcam (Cat# 120192). Methyl-B-Cyclodextrin (MBCD) was from Sigma-Aldrich (Cat# 332615). Filipin III, Dynasore, or MBCD were added to cultures at concentrations shown immediately after removing doxorubicin. BKM-120 was purchased from Apex Bio (Cat#A3015, Batch#2) and resuspended in DMSO for a stock concentration of 2.44 mM. GDC-0941 was purchased from Apex Bio (Cat#A8210, Batch#2) and resuspended in DMSO for a stock concentration of 1.95 mM. The drug/media were changed every 2 days until timelines were completed. pHrodo red labeling kit for phagocytosis was purchased from Sartorius (Cat#4649).

### Immunoblotting

Cell lines were treated and harvested as indicated in the figures. Lysates were prepared using TNESV buffer (1% NP-40, 50 mM Tris-HCl (pH 7.5), 100 mM NaCl, 2 mM EDTA, and 10 mM NaVO4), and 20 μg of total protein was separated by 10% SDS-PAGE and then transferred to nitrocellulose membranes using Bio-Rad reagents and apparatus. Immunoblotting was performed as previously described [4] for PIK3C2B (goat polyclonal, AF7249; R&D Systems), CLTC (rabbit monoclonal, 4796S (D3C6); Cell Signaling), and actin (mouse monoclonal, MA5-15739; Invitrogen). All antibodies were used at 1:1,000 dilution.

### Live-cell imaging

IncuCyte S3 using software version 2019B and IncuCyte Sx5 using software version2021B were utilized for live-cell visualization of GFP, mCherry, and miRFP-713 constructs in cells. Images were taken every 2 hours for at least 25 images per well at 20× magnification from day 3 to day 8 following doxorubicin treatment.

### Engulfment quantification

IncuCyte S3 and SX5 live cell imagers were used to quantify engulfment. Images were taken every 2 hours from the 3D-7D post-doxorubicin time point. Parameters were optimized to quantify images as follows: First, thresholds were set on predator cells for fluorescence intensity (to exclude background fluorescent areas) and size (large size restriction used to exclude dead cells). Once these constraints were set, we established settings for quantifying the number of engulfed prey cells. As the prey cells are made smaller and rounded when fully internalized by the predator cells, we adjusted size and circularity parameters to account for these circumstances. Cell outline area setting was adjusted from the default (1) on both predators and prey to exclude normally occurring cell–cell contacts. The areas of predator/prey overlap were then quantified as a function of the number of predator cells, yielding the percentage of predator cells engulfing at each time point. Finally, to minimize counting overlaps due to floating cellular debris and cells that were overtopped but never internalized, we averaged the counts from 3 × 2-hour time points, yielding an average percentage engulfment for each 6-hour time point. For MCF-7: Segmentation Adjustment: 1 (default), Red MCF7 Predators: Tophat radius set to 45 μm, Threshold (OCU): 0.4, Edge sensitivity at −35, Hole fill: 60 μm^2^, Pixel size: 5, Area inclusion: 160 to 20,000 μm^2^, Eccentricity maximum: 0.99; Green MCF7 Prey: Tophat radius set to 10 μm, Threshold (GCU): 0.15, Edge sensitivity at −25, Hole fill: 500 μm^2^, Pixel size: −3, Area minimum 10 μm^2^, Eccentricity inclusion 0.4 to 0.9. For MPE600, settings were the same except for the following: Predator pixel size was increased to 5 as they had less background cell/cell contact; Prey Tophat radius was increased to 10 μm; Prey threshold (GCU) was increased to 0.75; Prey pixel size was also increased to 2, Area: maximum set to 80 μm^2^. For 4226 cells, Segmentation Adjustment: 2, Red 4226 Predators: Tophat radius: 75 μm, Threshold (OCU): 0.15, Edge sensitivity at −40, Hole fill: 400 μm^2^, Pixel size to stop edge effect −5, Area inclusion: 324 to 20,000 μm^2^, Green 4226 Prey: Tophat radius set to 25 μm, Green threshold 2.5 GCU, Edge sensitivity at −35, Hole fill: 400 μm^2^, Pixel size to stop edge effect −3, Area inclusion 18 to 160 μm^2^, Eccentricity inclusion 0.3 to 0.96, Green threshold 2.5 GCU, Red + Green (Overlap) Parameters: Minimum Area: 5 μm. For each engulfment experiment, as a measure of predator viability over time, surface area of the predator cells was determined.

### Quantitative analysis of biosensor localization

MCF7 predator cells expressing both biosensor-GFP and LYN11-mCherry were mixed with MCF7 prey expressing miRFP713 and imaged on IncuCyte live-cell imager. Images at specific stages of engulfment were exported as separate channels (GFP-biosensor, Orange-Lyn11, NIR-prey).

The images were imported into ImageJ and converted to greyscale. ImageJ line tool was used to manually quantify PI(3)P (as in Fig 4B), and polygon tool was used for creating ROI for quantification of PI(4)P and PI(4,5)P_2_ (as in Figs 1I, 3D and 3E) ROI measurements from each cell were normalized to the lowest mean intensity (LMI) for the GFP channel from the same cell, producing enrichment for the GFP channel at the indicated locations (i.e., nonengulfing membrane, cytoplasm, ring forming at the prey cell, as shown in Fig 1I). Both biosensor-GFP and LYN11-mCherry intensities were independently normalized to their respective LMI within the cell. The normalized biosensor-GFP intensity was then divided by normalized LYN11-mCherry intensity, giving the overall enrichment of the biosensor species relative to LYN11. Examples of these regions are shown in Figs 1K (upper), 3D, 3E and 4C. To generate graphs for PI(3)P, 10 independent engulfing cells were quantified. For PI(4)P and PI(4,5)P2, 6 independent engulfing cells were quantified.

### pHrodo time-lapse quantification

pHrodo-labeling of prey cells was preformed (in accordance with manufacturer’s instructions) on MCF-7 cells prior to incubation with senescent MCF7 predator cells. In brief, MCF-7 prey cells were plated (2.5 million cells in 10 cm dish) and treated with 0.25 uM doxorubicin. Fresh media was added 24 hours later. After 3 days, MCF-7 prey cells were trypsinized and counted, 100,000 cells were washed with 1 mL of prewarmed “IncuCyte pHrodo wash buffer,” then briefly rinsed in 1 mL of prewarmed “IncuCyte pHrodo labeling buffer.” Cells were then spun down at 400 RCF × 5 min and resuspended in 1 mL of pHrodo red labeling dye at a concentration of 100 ng/ml in IncuCyte pHrodo labeling buffer and incubated at 37°C for 1 hour. Resulting pHrodo-labeled cells were pelleted, rinsed with PBS, resuspended in growth media, and plated on senescent (day 3 post-DOXO) MCF7-GFP cells at a ratio of 1 predator cell:4 prey cells. Quantification of biosensor-GFP predator cells engulfing pHrodo-labeled prey over time was preformed using ImageJ at time points centered around the point of greatest increase in pHrodo fluorescence between 2 time points over the time course. Three regions in the cytoplasm were measured and averaged for each image. The cell membrane and predator/prey contact points were manually outlined using the polygon tool and normalized to mean cytoplasm ROI intensity. Time course quantification was performed for 5 cells. Data points plotted represent mean intensity ratios for ROI indicated (e.g., ring intensity/cytoplasm intensity).

### Microscopy

Confocal images of fluorescent cells were captured on a Nikon TiE-2 Inverted Research Microscope Nikon A1rSi Laser Point Scanning Confocal, with a Plan Apo λ 60× oil objective for both cell lines and tissue sections. Image brightness and background levels were adjusted uniformly and minimally for entire images using NIS-Elements software or Photoshop when necessary for visual clarity in publication. In the case of experiments with miRFP713 (near-infrared) constructs, imaging was acquired in the Cy5 channel and spectrally unmixed in Nikon’s NIS elements software version 5.02.01 with default settings.

### Statistics

In all IncuCyte experiments with cellular engulfment quantified, 25 fields of view per well were taken for 3 wells of each treatment group and 3 wells of the control group every 2 hours. Two biological replicates of each experiment were performed. The percentages from 3 consecutive time points (6 hours) were then averaged. For IncuCyte experiments containing 2 conditions, unpaired *t* tests with 2-stage step-up (Benjamini, Krieger and Yekutieli) model was performed. A minimum of 2,000 predator cells were counted for each 2-hour time point. Engulfment percentages were compared for the final time point of the engulfment experiments. For IncuCyte experiments containing 3 or more conditions, 2-way ANOVA with a Dunnett’s post-test to correct for multiple comparisons was performed using Prism 9 software. Error bars indicate SEM. *P* values (*) < 0.05, (**) < 0.002, (***) < 0.0002, (****) < 0.0001.

## Supporting information

S1 Fig Filamentous actin in senescent 4226 mammary tumor predator cells localizes to prey cells throughout the engulfment process. Predator 4226 cell line expressing LifeAct-GFP were treated with 750 nM doxorubicin for 24 hours, washed, and plated on untreated mCherry 4226 prey cells. On day 7 post-doxorubicin, cells were fixed, DAPI stained, and imaged on the confocal microscope. (**A**) Axial slices of a predator cell that has partially overtopped a prey cell. Dashed arrows indicate LifeAct-GFP localization. (**B**) Axial slices of a predator cell that has partially overtopped a prey cell. Closed arrows indicate LifeAct-GFP localization. (**C**) Axial slices of a predator cell that has engulfed and broken down a prey cell. Open arrows indicate LifeAct-GFP localization. Scale bar = 10 μm.
(PDF)

S2 Fig PI biosensors expressed in predator 4226 cells reveal PI(4,5)P2, PI(4)P, and PI(3)P localize to the prey cells during engulfment. 4226 mammary tumor cell lines expressing biosensors that detect indicated PI species were treated with 750 nM doxorubicin for 24 hours, washed, and plated on untreated mCherry-4226 “prey” cells. Cultures were imaged over days 3–8. Time course live-cell imaging of senescent 4226 cells that express (**A**) PLCD1-GFP marking PI(4,5)P2; (**B**) P4M-SidMx2-GFP marking PI(4)P; (**C**) 2xFYVE-GFP marking PI(4)P; (**D**) PLCD1(R40L)-GFP (Negative Ctrl) mutant that does not bind PI species, throughout the entire process of engulfing mCherry-4226 cells. Scale bar = 100 μm.
(PDF)

S3 Fig Model of PI species localization during the 5 stages of engulfment. During early stages of contact and overtopping, predator PI(4)P and PI(4,5)P2 are highly localized to prey cells and remain concentrated at rounding. PI(3)P is localized at rounding, internalization, and is present during early stages of digestion before dissipating.
(PDF)

S4 Fig *PI3C2B* but not *PIK3C2A*, *PIK3C3*, *PIK3C2G* is required for engulfment. (**A**) In MCF-7 cells, CRISPR-Cas9 knockout of *PIK3C2A*, *PIK3C3*, *PIK3C2G* single-cell clones were screened by immunoblot as indicated in the figure. Clones indicated by asterisk were chosen for further testing. (**B**) Predator cell engulfment rates (upper) and confluency as a measure of viability (lower) for senescent MCF-7 parental cells and indicated knockout clones were determined by time course imaging in IncuCyte. Underlying data can be found at S1 Data. (**C**) CRISPR-Cas9 single-cell clone Pik3c2b knockouts of 4226 cells were screened by immunoblot. (**D**) Predator cell engulfment rates (left) and confluency (right) of senescent 4226 parental cells and 3 Pik3c2b knockout clones were determined by time course imaging. Underlying data can be found at S1 Data. (**E**, **F**) Time course live-cell imaging of a senescent MCF-7 cell expressing mCherry (**E**) or MCF-7-PIK3C2B-KO knockout (**F**) throughout the entire process of engulfing MCF-7-GFP untreated (NT) cells. Open arrows indicate successful engulfment by a senescent MCF-7 predator cell; closed arrows show representative failed engulfment by MCF-7-PIK3C2B-KO cells. Scale bar = 100 μm.
(PDF)

S5 Fig Inhibition of PI3 kinase does not reduce engulfment rates. (**A**-**D**) MCF-7 (**A**) and 4226 (**B**) cells were made senescent by doxorubicin treatment, then treated with the indicated selective PI3K inhibitor and cell viability was determined by MTT assay. Underlying data can be found at S1 Data. (**C**, **D**) Mixed cultures of mCherry and GFP expressing cells were made senescent by doxorubicin to generate senescent predator/senescent prey cultures (DOXO-DOXO cultures), and engulfment rates (upper graphs) and confluency as a measure of viability (lower graphs) were determined for MCF-7 (**C**) and 4226 cells (**D**) treated with the PI3 kinase inhibitor as indicated in the figure. Underlying data can be found at S1 Data.
(PDF)

S6 Fig Visualization of IncuCyte parameters for quantification of engulfment rates. MCF-7 predator cells were treated with 250 nM doxorubicin for 24 hours, washed, and plated on untreated mCherry-MCF-7 “prey” cells. Cultures were imaged over days 4–8. Upper displays unmasked predator (GFP) and prey (mCherry) cells during engulfment. Lower shows a representative imaging mask used for quantification of cell-in-cell. Pink masking is used for predator cells, while blue masking is used for prey cells. Overlap of the 2 masks is indicated by white mask in the final frame. Cells are not counted as “engulfed” until prey are in a rounded and fully circumscribed state of overlap. Closed arrows indicate a cell negative for engulfment; open arrows indicate a cell counted as a positive.
(PDF)

S7 Fig Clathrin is required for efficient completion of internalization. (**A**) Predator cell engulfment rates (upper) and confluency (lower) were determined for senescent 4226-GFP cells that were treated with indicated drugs. Underlying data can be found at S1 Data. (**B**) Predator cell engulfment rates (upper) and confluency (lower) were determined for MCF-7 senescent predator/senescent prey cells (“DOXO-DOXO” cultures) that were treated with indicated drugs. Underlying data can be found at S1 Data. (**C**) Immunoblot verification of shRNA knockdown of CLTC in MCF-7 and MPE600 cells as indicated. Actin loading control is shown in lower panels. (**D**) Predator cell engulfment rates (upper) and confluency (lower) were determined for senescent GFP-MPE600 cells that were made to express an shRNA construct targeting CLTC. Underlying data can be found at S1 Data.
(PDF)

S1 Data Underlying data for Figs 1J, 3D–3F, 4B, 4D, 6B, 7B, S4B, S4D, S5A–S5D, S7A, S7B and S7D. (XLSX)

S1 Raw images The uncropped immunoblots for Figs 6A, S4A, S4C and S7C. (PDF)

S1 Video Corresponds to Fig 1A. (**A**) Time lapse of confocal imaging and volume view reconstruction of doxorubicin-induced senescent MCF-7-LifeAct cell engulfing multiple untreated MCF-7 mCherry cells over the course of 6.75 hours with 11 z-planes taken at a spacing of 1.6 μm per plane. Video was recorded at 4 frames per hour. (**B**) The same video as in (**A**), except the top part of the predator cell above the axial plane was removed to visualize cytoplasmic actin.
(AVI)

S2 Video Corresponds to Fig 1B. (**A**, **B**) Time lapse of confocal imaging and volume view reconstructions of 1 doxorubicin-induced senescent MCF-7-LifeAct-GFP cell that has engulfed and is breaking down multiple untreated MCF-7 mCherry cells over the course of 9.75 hours with 17 z-planes taken at a spacing of 1.6 μm per plane, at 4 frames per hour. (**A**) The same video is shown with the top 51.2% of the axial plane of the cell removed to show interior actin concentration and actin localization upon digestion. (**B**) Volume view of entire cell.
(AVI)

S3 Video Corresponds to Fig 1C. Confocal imaging and animation of volume view reconstruction of doxorubicin-induced senescent MCF-7-LifeAct-GFP cell contacting and overtopping an untreated MCF-7 mCherry cell.
(AVI)

S4 Video Corresponds to Fig 1D. Confocal imaging and animation of volume view reconstruction of doxorubicin-induced senescent MCF-7-LifeAct-GFP cell that has overtopped an untreated MCF-7 mCherry cell.
(AVI)

S5 Video Corresponds to Fig 1E. Confocal imaging and animation of volume view reconstruction of doxorubicin-induced senescent MCF-7-LifeAct-GFP cell rounding and beginning to internalize an untreated MCF-7 mCherry cell.
(AVI)

S6 Video Corresponds to Fig 3A (lower). Confocal imaging and animation of volume view reconstruction of doxorubicin-induced senescent MCF-7-PLCD1-GFP cell that has partially engulfed an untreated MCF-7 mCherry cell.
(AVI)

S7 Video Corresponds to Fig 3B (upper). Confocal imaging and animation of volume view reconstruction of doxorubicin-induced senescent MCF-7-P4M-SidMx2-GFP cell that has partially engulfed an untreated MCF-7 mCherry cell.
(AVI)

S8 Video Corresponds to Fig 3C (upper). (**A**) Confocal imaging and animation of volume view reconstruction of doxorubicin-induced senescent MCF-7-2xFYVE-GFP cell that has internalized an untreated MCF-7 mCherry cell. Corresponds to Fig 3C (lower). (**B**) Zoomed-in volume view reconstruction of the same 2xFYVE-GFP structure.
(AVI)

## Abbreviations

CME: Clathrin-mediated endocytosis
LMI: lowest mean intensity
NT: not-treated
PI: phosphoinositide
SASP: senescence-associated secretory phenotype
