# Supplementary figures and images for: Phosphoinositide species and filamentous actin formation mediate engulfment by senescent tumor cells
Source: PLoS Biol. 2022 Oct 24;20(10):e3001858. doi: 10.1371/journal.pbio.3001858 (PMC9632905; doi:10.1371/journal.pbio.3001858)

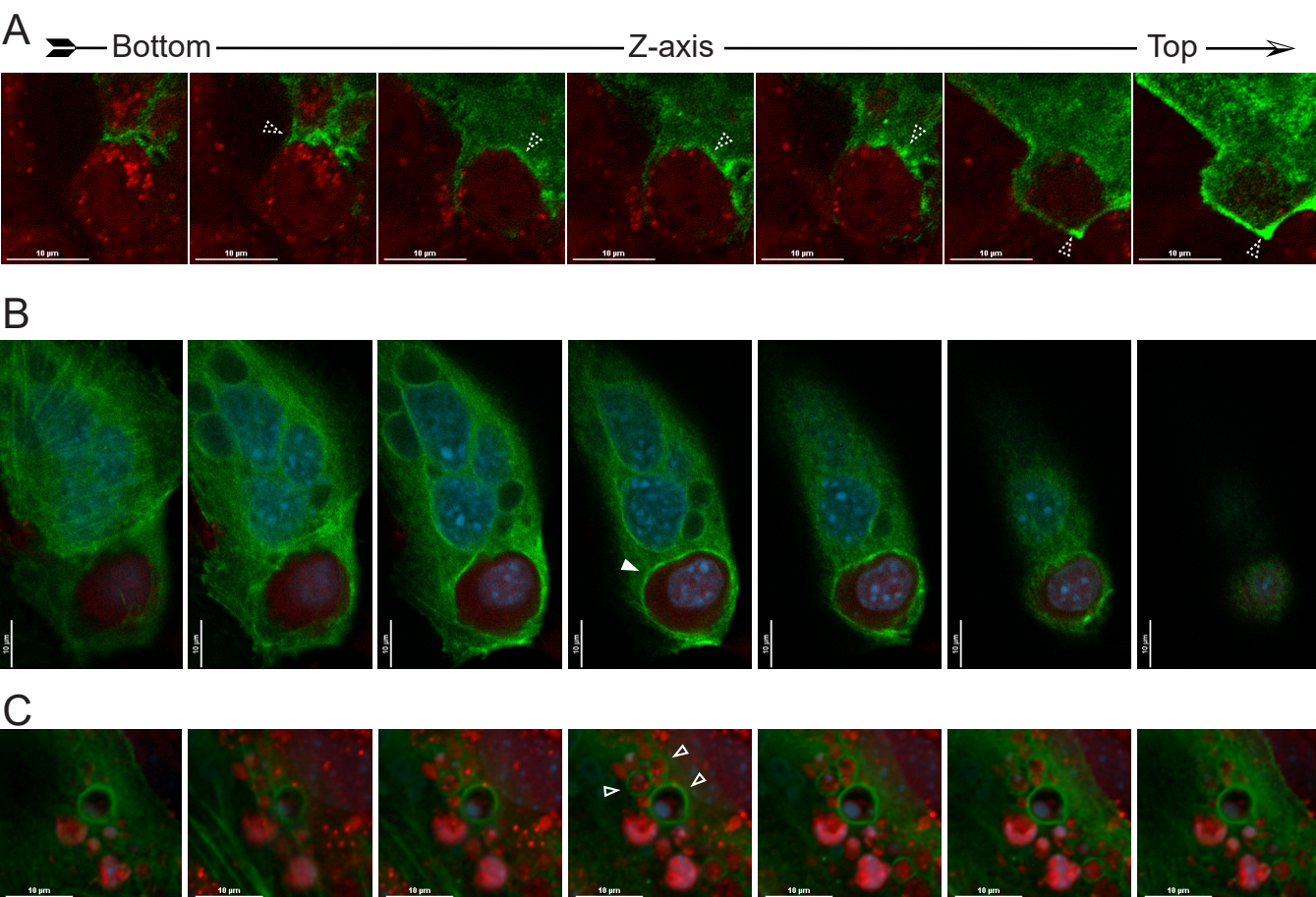

Supplement: S1 Fig — Predator 4226 cell line expressing LifeAct-GFP were treated with 750 nM doxorubicin for 24 hours, washed, and plated on untreated mCherry 4226 prey cells. On day 7 post-doxorubicin, cells were fixed, DAPI stained, and imaged on the confocal microscope. (A) Axial slices of a predator cell that has partially overtopped a prey cell. Dashed arrows indicate LifeAct-GFP localization. (B) Axial slices of a predator cell that has partially overtopped a prey cell. Closed arrows indicate LifeAct-GFP localization. (C) Axial slices of a predator cell that has engulfed and broken down a prey cell. Open arrows indicate LifeAct-GFP localization. Scale bar = 10 μm. (PDF) [file pbio.3001858.s001.pdf]

Supplemental Figure S2

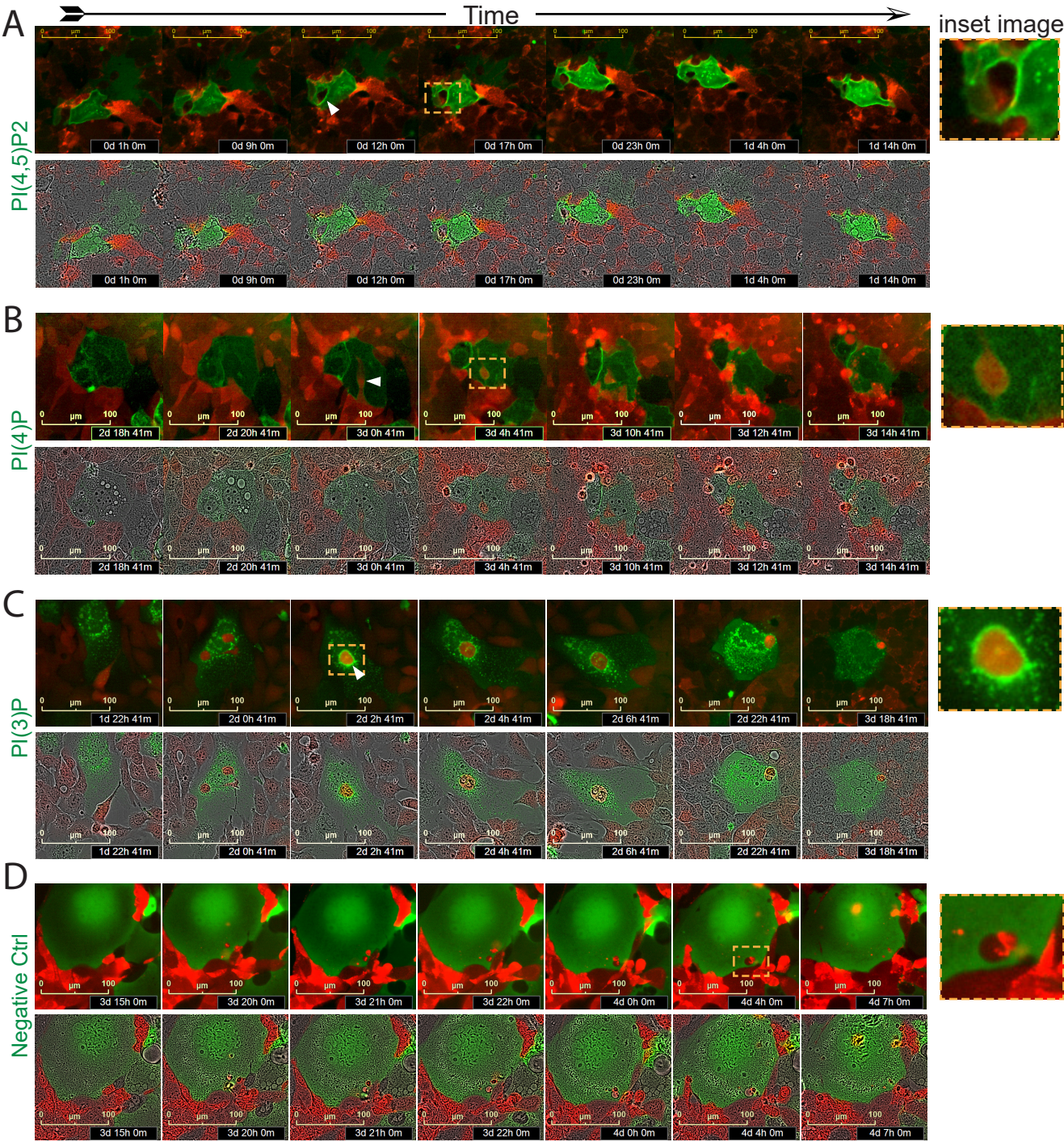

Supplement: S2 Fig — 4226 mammary tumor cell lines expressing biosensors that detect indicated PI species were treated with 750 nM doxorubicin for 24 hours, washed, and plated on untreated mCherry-4226 “prey” cells. Cultures were imaged over days 3–8. Time course live-cell imaging of senescent 4226 cells that express (A) PLCD1-GFP marking PI(4,5)P2; (B) P4M-SidMx2-GFP marking PI(4)P; (C) 2xFYVE-GFP marking PI(4)P; (D) PLCD1(R40L)-GFP (Negative Ctrl) mutant that does not bind PI species, throughout the entire process of engulfing mCherry-4226 cells. Scale bar = 100 μm. (PDF) [file pbio.3001858.s002.pdf]

Supplemental Figure S3

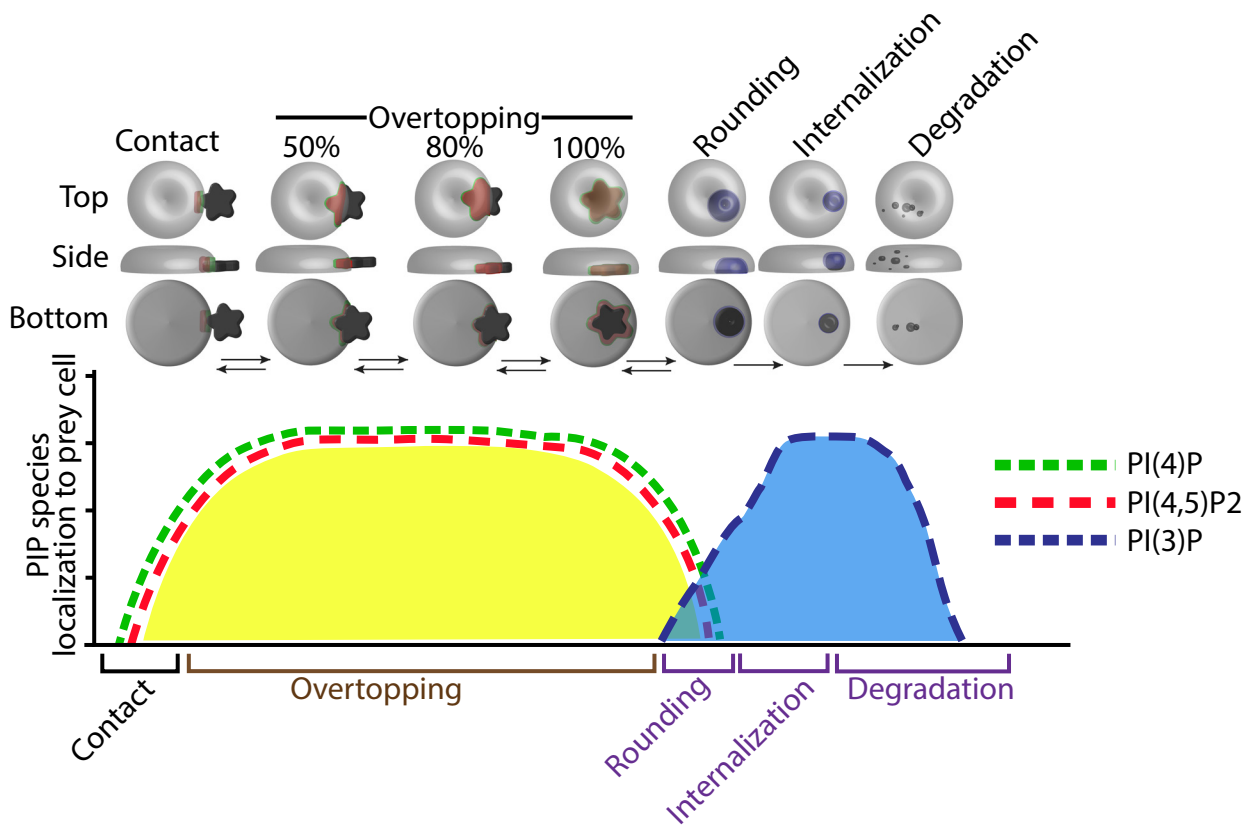

Supplement: S3 Fig — During early stages of contact and overtopping, predator PI(4)P and PI(4,5)P2 are highly localized to prey cells and remain concentrated at rounding. PI(3)P is localized at rounding, internalization, and is present during early stages of digestion before dissipating. (PDF) [file pbio.3001858.s003.pdf]

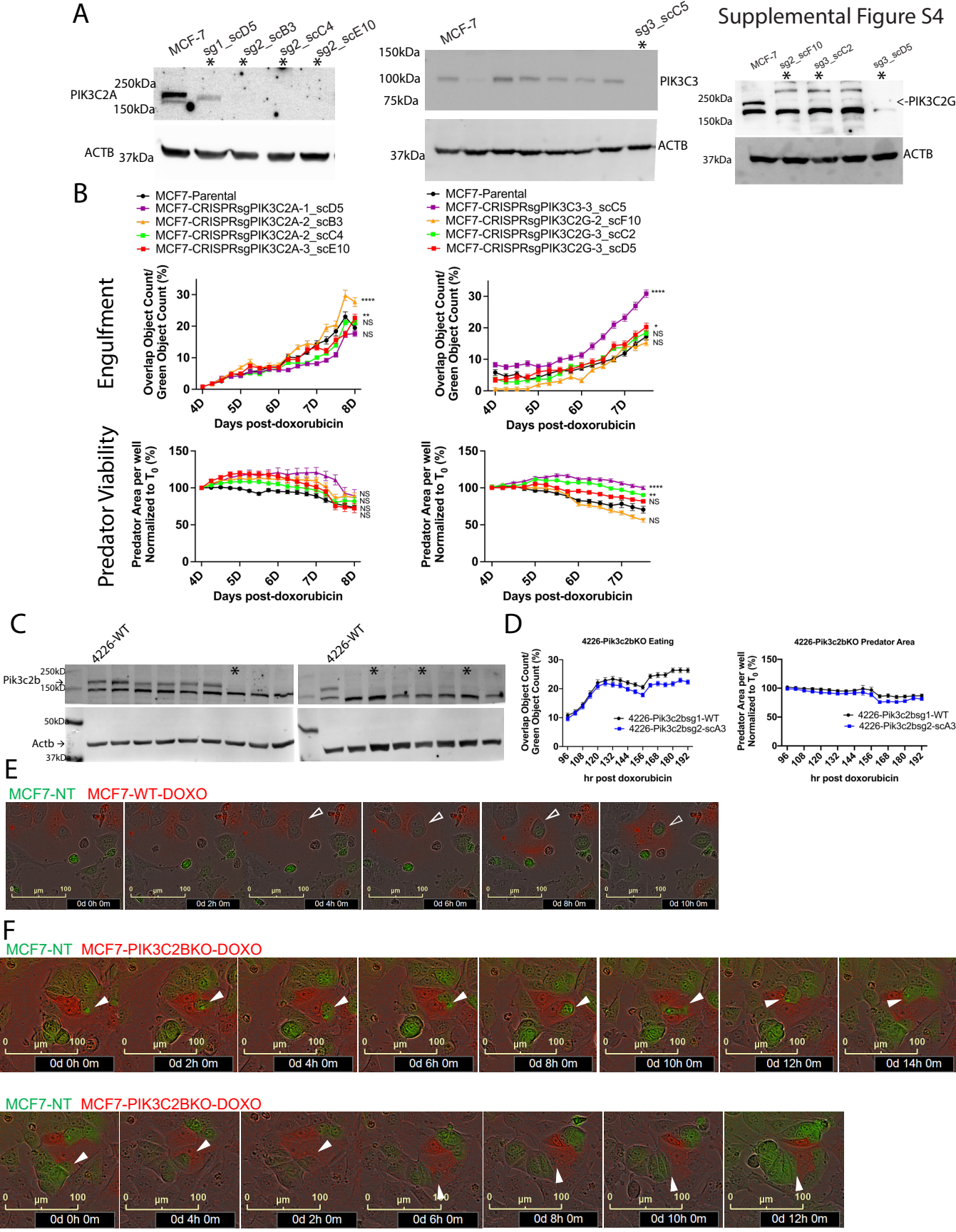

Supplement: S4 Fig — (A) In MCF-7 cells, CRISPR-Cas9 knockout of PIK3C2A, PIK3C3, PIK3C2G single-cell clones were screened by immunoblot as indicated in the figure. Clones indicated by asterisk were chosen for further testing. (B) Predator cell engulfment rates (upper) and confluency as a measure of viability (lower) for senescent MCF-7 parental cells and indicated knockout clones were determined by time course imaging in IncuCyte. Underlying data can be found at S1 Data. (C) CRISPR-Cas9 single-cell clone Pik3c2b knockouts of 4226 cells were screened by immunoblot. (D) Predator cell engulfment rates (left) and confluency (right) of senescent 4226 parental cells and 3 Pik3c2b knockout clones were determined by time course imaging. Underlying data can be found at S1 Data. (E, F) Time course live-cell imaging of a senescent MCF-7 cell expressing mCherry (E) or MCF-7-PIK3C2B-KO knockout (F) throughout the entire process of engulfing MCF-7-GFP untreated (NT) cells. Open arrows indicate successful engulfment by a senescent MCF-7 predator cell; closed arrows show representative failed engulfment by MCF-7-PIK3C2B-KO cells. Scale bar = 100 μm. (PDF) [file pbio.3001858.s004.pdf]

A

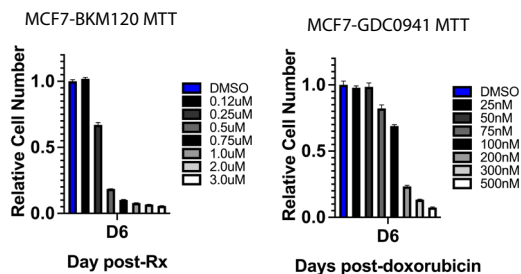

B

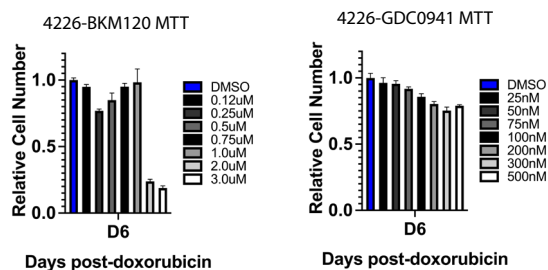

C

Engulfment

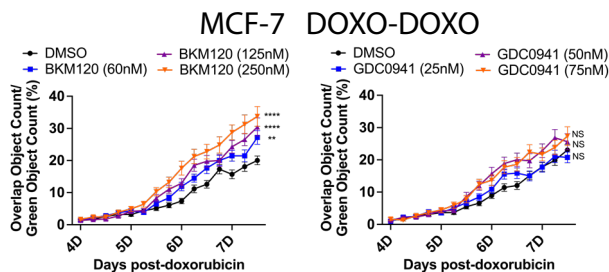

D

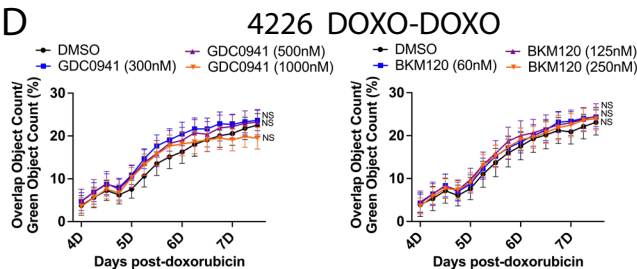

Predator Viability

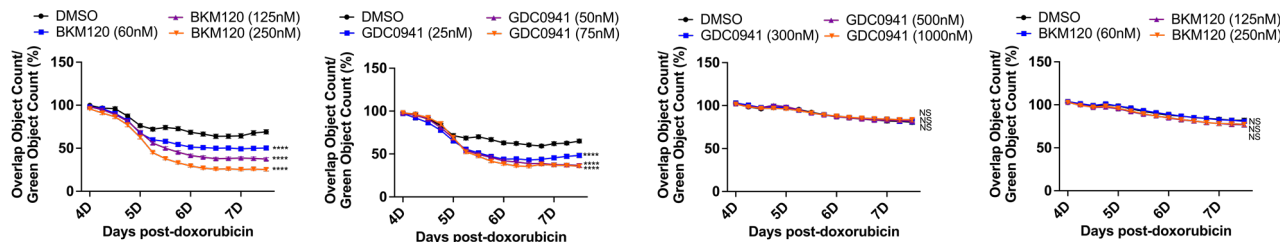

Supplement: S5 Fig — (A-D) MCF-7 (A) and 4226 (B) cells were made senescent by doxorubicin treatment, then treated with the indicated selective PI3K inhibitor and cell viability was determined by MTT assay. Underlying data can be found at S1 Data. (C, D) Mixed cultures of mCherry and GFP expressing cells were made senescent by doxorubicin to generate senescent predator/senescent prey cultures (DOXO-DOXO cultures), and engulfment rates (upper graphs) and confluency as a measure of viability (lower graphs) were determined for MCF-7 (C) and 4226 cells (D) treated with the PI3 kinase inhibitor as indicated in the figure. Underlying data can be found at S1 Data. (PDF) [file pbio.3001858.s005.pdf]

Supplemental Figure S6

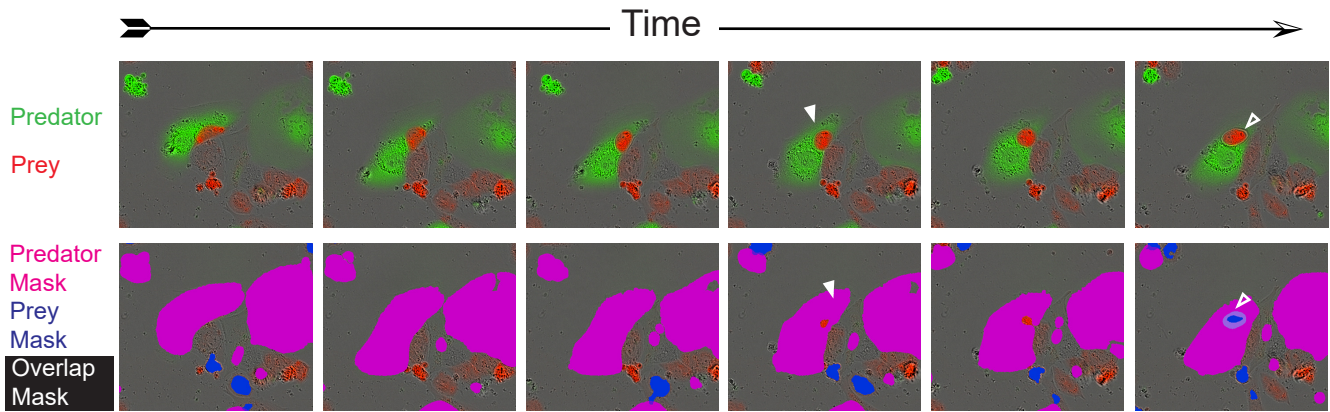

Supplement: S6 Fig — MCF-7 predator cells were treated with 250 nM doxorubicin for 24 hours, washed, and plated on untreated mCherry-MCF-7 “prey” cells. Cultures were imaged over days 4–8. Upper displays unmasked predator (GFP) and prey (mCherry) cells during engulfment. Lower shows a representative imaging mask used for quantification of cell-in-cell. Pink masking is used for predator cells, while blue masking is used for prey cells. Overlap of the 2 masks is indicated by white mask in the final frame. Cells are not counted as “engulfed” until prey are in a rounded and fully circumscribed state of overlap. Closed arrows indicate a cell negative for engulfment; open arrows indicate a cell counted as a positive. (PDF) [file pbio.3001858.s006.pdf]
