# Supplementary material for: Phosphoinositide species and filamentous actin formation mediate engulfment by senescent tumor cells
Source: PLoS Biol. 2022 Oct 24;20(10):e3001858. doi: 10.1371/journal.pbio.3001858 (PMC9632905; doi:10.1371/journal.pbio.3001858)

Supplemental Figure S7

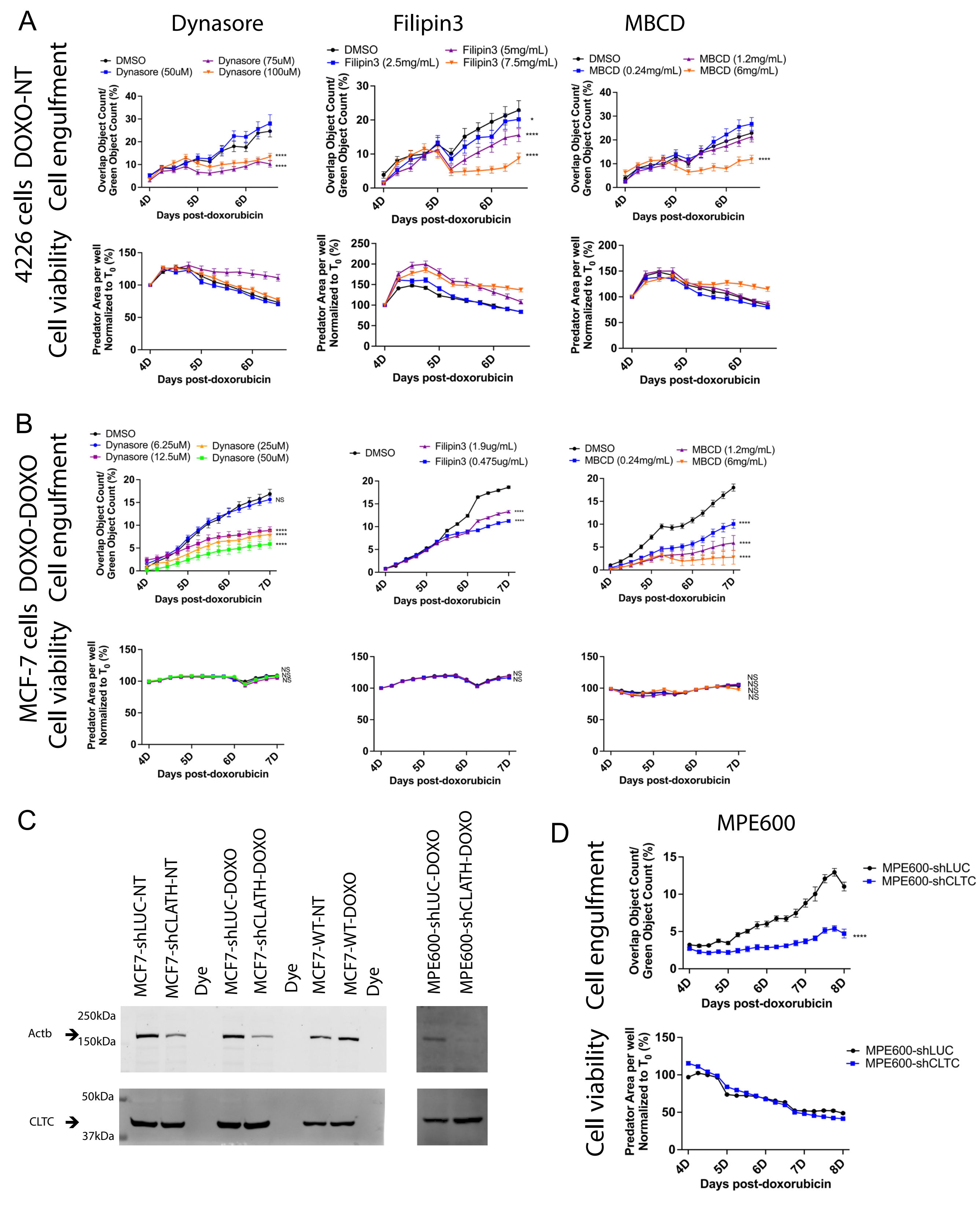

Supplement: S7 Fig — (A) Predator cell engulfment rates (upper) and confluency (lower) were determined for senescent 4226-GFP cells that were treated with indicated drugs. Underlying data can be found at S1 Data. (B) Predator cell engulfment rates (upper) and confluency (lower) were determined for MCF-7 senescent predator/senescent prey cells (“DOXO-DOXO” cultures) that were treated with indicated drugs. Underlying data can be found at S1 Data. (C) Immunoblot verification of shRNA knockdown of CLTC in MCF-7 and MPE600 cells as indicated. Actin loading control is shown in lower panels. (D) Predator cell engulfment rates (upper) and confluency (lower) were determined for senescent GFP-MPE600 cells that were made to express an shRNA construct targeting CLTC. Underlying data can be found at S1 Data. (PDF) [file pbio.3001858.s007.pdf]
