# Supplementary material for: Phosphoinositide species and filamentous actin formation mediate engulfment by senescent tumor cells
Source: PLoS Biol. 2022 Oct 24;20(10):e3001858. doi: 10.1371/journal.pbio.3001858 (PMC9632905; doi:10.1371/journal.pbio.3001858)

Fig. 6A

MCF7-PIK3C2BKOs
PIK3C2B detected with A488 secondary antibody

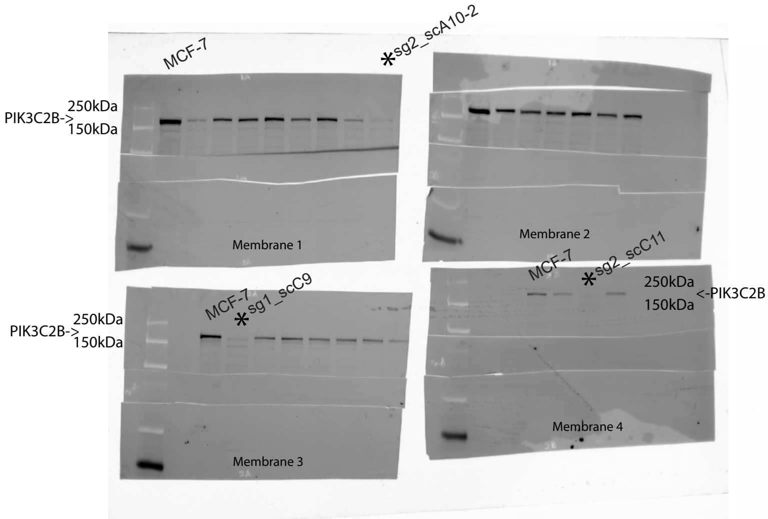

WB: PIK3C2B
Fig. 6A

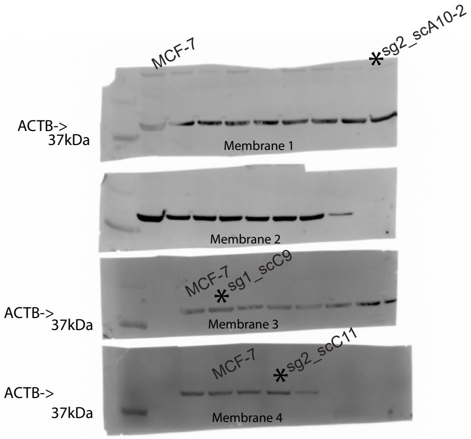

WB: ACTB
Fig. 6A

Supp Fig. 7C

MCF7-shCLTC
CLTC detected with A800 secondary antibody

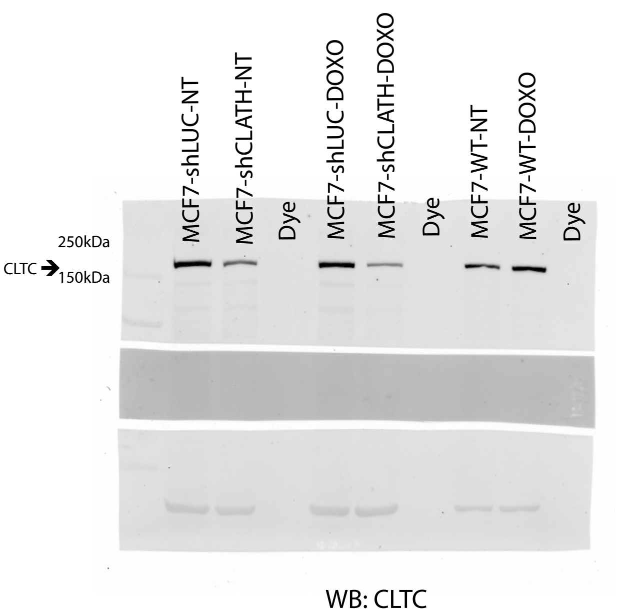

WB: CLTC
Supp Fig. 7C

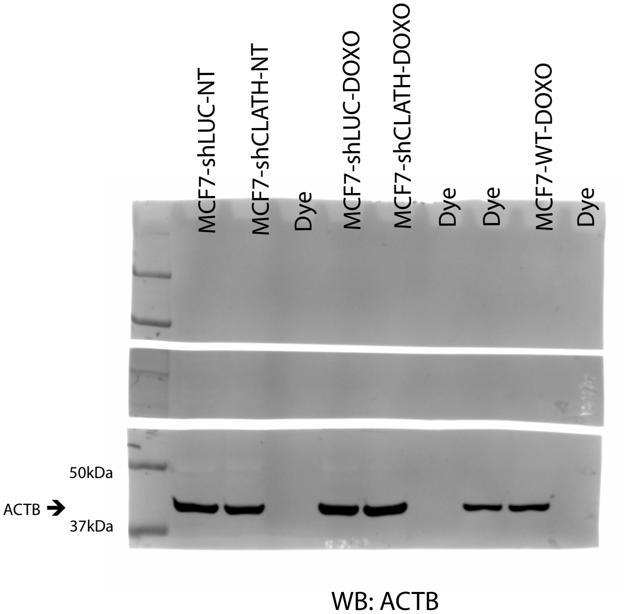

WB: ACTB
Supp Fig. 7C

MPE600-shCLTC
CLTC detected with A800 secondary antibody

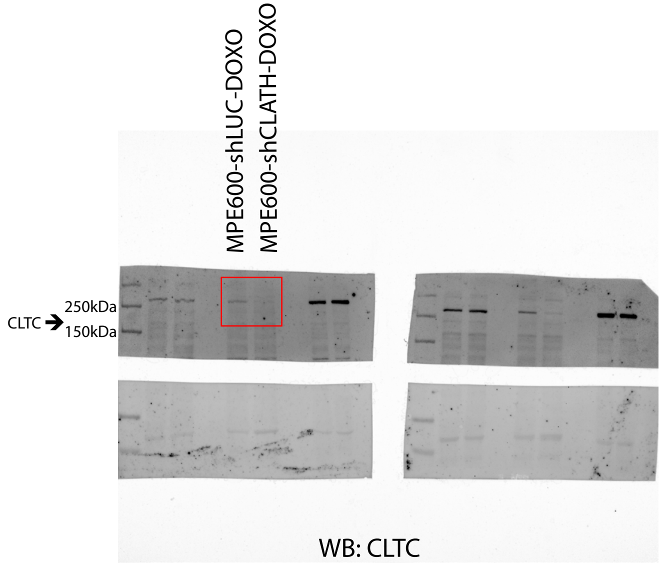

WB: CLTC
Supp Fig. 7C

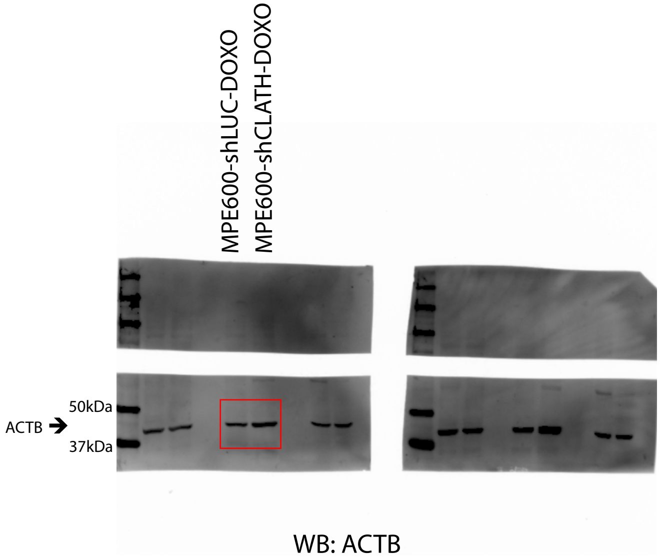

WB: ACTB
Supp Fig. 7C

Supp Fig 4A

MCF7-PIK3C2AKOs
PIK3C2A detected with HRP secondary antibody

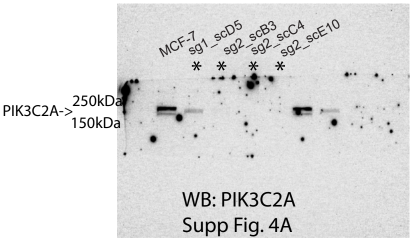

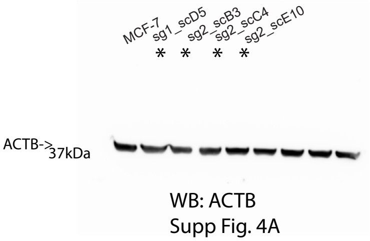

MCF7-PIK3C3KOs
PIK3C3 detected with HRP secondary antibody

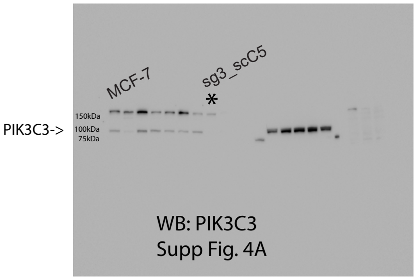

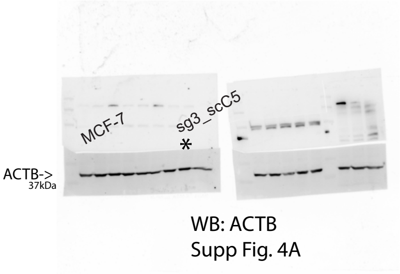

MCF7-PIK3C2GKOs
PIK3C2G detected with HRP secondary antibody

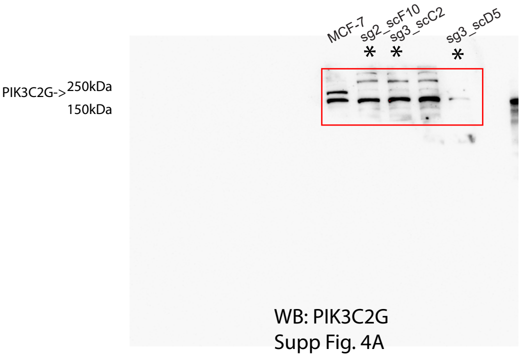

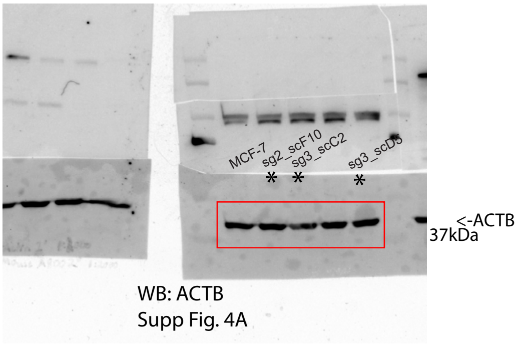

Supp Fig 4C

4226-Pik3c2bKOs
Pik3c2b detected with A800 secondary antibody

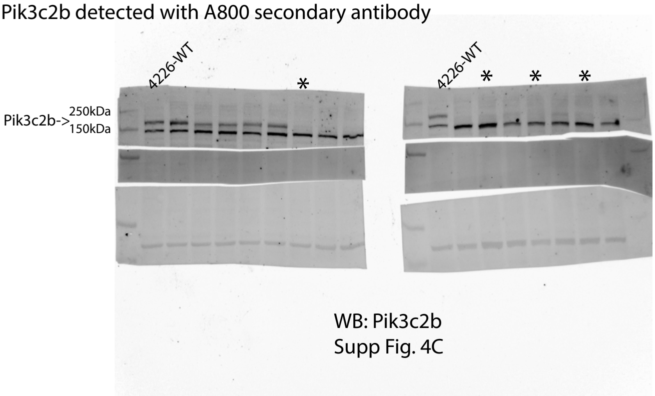

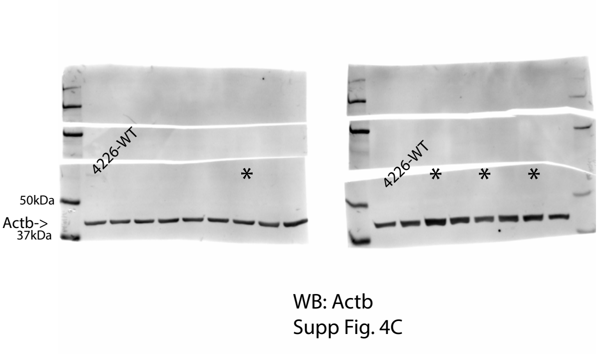

Supplement: S1 Raw images — (PDF) [file pbio.3001858.s009.pdf]
